# Light-driven biohybrid system utilizes N_2_ for photochemical CO_2_ reduction

**DOI:** 10.1093/nsr/nwad142

**Published:** 2023-05-15

**Authors:** Jin-Yue Zeng, Xiao-Shuang Wang, Xin-Hua Liu, Qian-Ru Li, Jun Feng, Xian-Zheng Zhang

**Affiliations:** Key Laboratory of Biomedical Polymers of Ministry of Education, and Department of Chemistry, Wuhan University, Wuhan 430072, China; Key Laboratory of Biomedical Polymers of Ministry of Education, and Department of Chemistry, Wuhan University, Wuhan 430072, China; Key Laboratory of Biomedical Polymers of Ministry of Education, and Department of Chemistry, Wuhan University, Wuhan 430072, China; Key Laboratory of Biomedical Polymers of Ministry of Education, and Department of Chemistry, Wuhan University, Wuhan 430072, China; Key Laboratory of Biomedical Polymers of Ministry of Education, and Department of Chemistry, Wuhan University, Wuhan 430072, China; Key Laboratory of Biomedical Polymers of Ministry of Education, and Department of Chemistry, Wuhan University, Wuhan 430072, China; Wuhan Research Centre for Infectious Diseases and Cancer, Chinese Academy of Medical Sciences, Wuhan 430071, China

**Keywords:** biohybrid, photocatalysis, CO_2_ reduction, nitrogen fixation, bacterium

## Abstract

Attempting to couple photochemical CO_2_ reduction with N_2_ fixation is usually difficult, because the reaction conditions for these two processes are typically incompatible. Here, we report that a light-driven biohybrid system can utilize abundant, atmospheric N_2_ to produce electron donors via biological nitrogen fixation, to achieve effective photochemical CO_2_ reduction. This biohybrid system is constructed by incorporating molecular cobalt-based photocatalysts into N_2_-fixing bacteria. It is found that N_2_-fixing bacteria can convert N_2_ into reductive organic nitrogen and create a localized anaerobic environment, which allows the incorporated photocatalysts to continuously perform photocatalytic CO_2_ reduction under aerobic conditions. Specifically, the light-driven biohybrid system displays a high formic acid production rate of over 1.41 × 10^−14^ mol h^−1^ cell^−1^ under visible light irradiation, and the organic nitrogen content undergoes an over-3-fold increase within 48 hours. This work offers a useful strategy for coupling CO_2_ conversion with N_2_ fixation under mild and environmentally benign conditions.

## Teaser

Light-driven biohybrid system was created for achieving effective photochemical CO_2_ reduction by utilizing abundant, atmospheric N_2_ via biological nitrogen fixation to produce electron donors.

## INTRODUCTION

The coupling of CO_2_ conversion and N_2_ fixation is important for addressing current energy and environmental issues and achieving carbon neutrality [1,2]. On the one hand, rising atmospheric CO_2_, due to the burning of fossil fuels, is one of the major causes of global warming [3]. While recent advances in photocatalytic materials for CO_2_ conversion enable high photoconversion efficiency and broadband light absorption, a usually overlooked necessity for most state-of-the-art photocatalysts is the requirement of a sacrificial electron donor, typically an electron-rich reductive organic amine [4,5]. This consumption of sacrificial reagents constitutes a major economic and environmental disincentive for exploiting photochemical CO_2_ reduction in the search for sustainable energy sources. On the other hand, the Haber-Bosch (H-B) process is still the main method of artificial N_2_ fixation, and demands high reaction pressures (∼100 atm) and temperatures (∼700 K) and large amounts of hydrogen gas (H_2_) [6]. The source of H_2_ is often natural gas, and thus the H-B process produces ∼1.9 tons of CO_2_ per ton of NH_3_ generated (3 CO_2_ per 8 NH_3_) [5]. Nonetheless, the intrinsic chemical inertness of CO_2_ (C=O, 806 kJ mol^−1^) and N_2_ (N≡N, 940.95 kJ mol^−1^) inevitably impede the activation of CO_2_/N_2_ conversion [7,8]. The conventional thermodynamic coupling of CO_2_ and N_2_ fixation is typically energy intensive and requires harsh reaction conditions [9]. To this end, much effort has been devoted to transforming CO_2_ and N_2_ into value-added products under mild reaction conditions [10]. For example, the electrocatalytic coupling of CO_2_ and N_2_ conversion to produce urea has been explored by using inorganic catalysts such as PdCu/TiO_2_ hybrids, Bi-BiVO_4_ heterostructures and Ni_3_(BO_3_)_2_ nanocrystals [11–13]. To date, the electrochemical approach can realize the coupling of CO_2_ and N_2_ conversion under ambient conditions, but this process is electricity dependent and generally suffers from low Faradaic efficiency [12]. Compared to electrocatalytic approaches, photosynthesis could provide an appealing route for coupling CO_2_ reduction and N_2_ fixation under mild conditions by using solar energy [14]. Currently, photocatalytic materials for the coupling of CO_2_ reduction and N_2_ fixation are rarely reported. Although some inorganic photocatalysts such as Zn-doped MIL-88A and TiO_2_ have been investigated for CO_2_ reduction and the conversion of N_2_ to NH_3_, reaction processes such as CO_2_ reduction and N_2_ fixation are usually competitive in these photocatalytic systems [15,16].

Photosynthetic biohybrid systems can integrate the photochemical properties of artificial photocatalysts with the synthetic potential of biological cells, which have attracted significant attention for CO_2_ reduction and enhancing N_2_ fixation [17–23]. Among these biological units, microorganisms are studied extensively for the construction of photosynthetic biohybrids because of their rapid proliferation potential and ability to convert renewable substrates into value-added products through genetically programmable multistep catalysis [24–28]. Recently, microorganism-based biohybrid systems have been investigated with regard to improving the N_2_ fixation and Calvin cycle-based CO_2_ conversion of microorganisms, and thus enhancing the accumulation of biomass [29,30]. However, in the Calvin cycle process, CO_2_ stems from the decomposition of organic acid rather than the environment. To date, a study on biohybrid systems for the coupling of photocatalytic CO_2_ reduction and N_2_ fixation has not been reported, and many different kinds of N_2_-fixing microorganisms and their possible integration with photocatalytic materials remain largely unexplored. Although photosynthetic biohybrid systems have the potential to achieve the coupling of CO_2_ reduction and N_2_ fixation, the integration of N_2_-fixing microorganisms and artificial photocatalytic systems has intrinsic challenges. For example, in the reported biohybrid systems, the photosensitized materials are generally inorganic semiconducting nanomaterials such as CdS, TiO_2_ and InP, but leaching of toxic metals as well as reactive-oxygen-species (ROS) generation present an engineering challenge [31,32]. In addition to the carbon source required for microorganism growth, a usually overlooked necessity for most photosynthetic biohybrid systems is the requirement of an external sacrificial electron donor, typically an amino acid [33]. Attempts to directly utilize the amino acid molecules generated from N_2_-fixing bacteria pose their own set of challenges, as N_2_-fixing bacteria prioritize the optimization of survival strategies rather than organic nitrogen production efficiencies [31–33]. Moreover, most N_2_-fixing microorganisms can convert N_2_ into bioavailable ammonia, but often inhibit the nitrogen fixation process in the presence of nitrate or a high concentration of ammonium ions (>10 mmol L^−1^) [34,35]. For instance, 10 mmol L^−1^ of ammonium ions inhibited the expression of nitrogenase genes in *Azotobacter vinelandii*, which is widely used for biohybrid system fabrication [36]. Some photosynthetic products such as O_2_, H_2_, CO and CH_4_ often restrain the nitrogenase activity of N_2_-fixing microorganisms [37–39].

**Figure 1.**
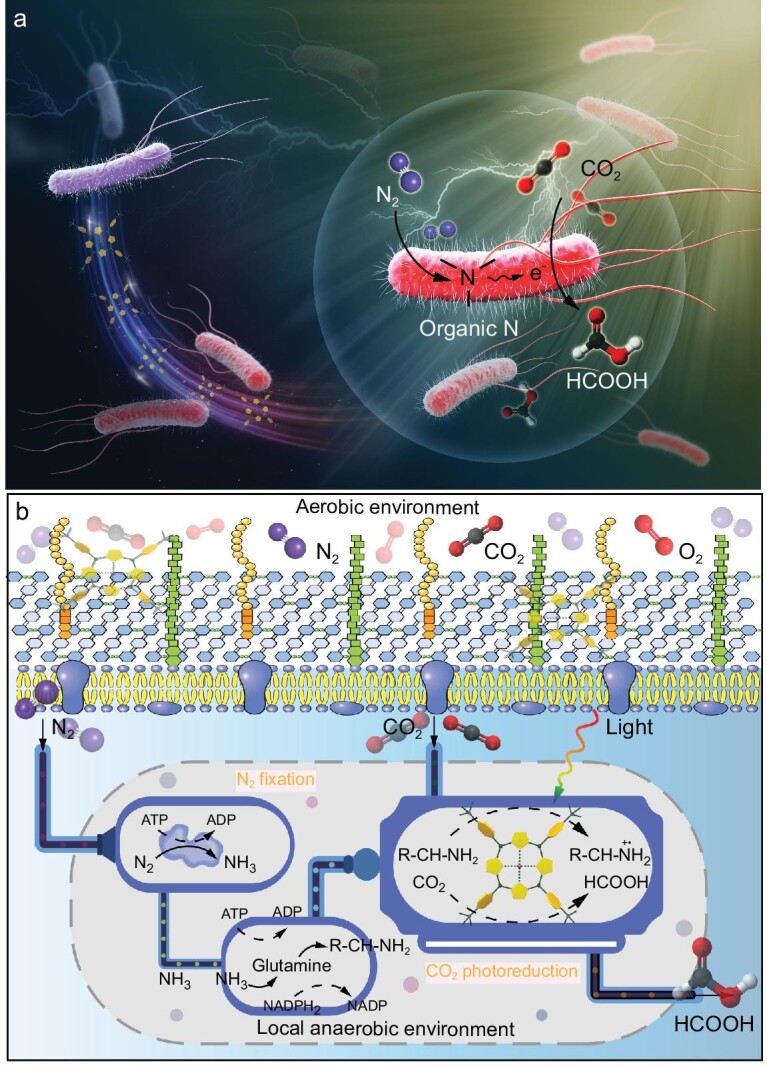
Schematic diagram of the light-driven biohybrid system. (a) Cobalt-based photocatalyst Co-TTAP was incorporated into bare N_2_-fixing bacteria to form N_2_-fixing bacteria/Co-TTAP biohybrid. The light-driven biohybrid system can utilize abundant, atmospheric N_2_ to generate organic nitrogen for achieving effective photochemical CO_2_ reduction. (b) Schematic of Co-bacteria. N_2_-fixing bacteria can utilize N_2_ to produce reduced organic nitrogen as electron donor and create a local anaerobic environment, which allows the biohybrid system to continue photochemical CO_2_ reduction aerobically.

Here, a light-driven biohybrid system was constructed for coupling photocatalytic CO_2_ reduction with biological N_2_ fixation by incorporating molecular organic cobalt-based photocatalysts into non-photosynthetic N_2_-fixing bacteria (Fig. 1a). The utilized N_2_-fixing bacterium *Paenibacillus azotofixans* (*P. azotofixans*) is Gram-positive, spore forming and facultative anaerobic, and has a capacity to fix atmospheric N_2_ that is greater than the capacities for all other *Paenibacillus* species [40,41]. *P. azotofixans* can tolerate extreme environments and interact with many plants [42]. In contrast to other N_2_-fixing microorganisms, the transcription activation of nitrogenase genes in *P. azotofixans* is not affected in the presence of nitrate or a high concentration of ammonium ions (>60 mmol L^−1^) [43,44]. Furthermore, the resulting biohybrid system can fix N_2_ into bioavailable ammonia that can be utilized by *P. azotofixans* for incorporation into reductive organic nitrogen compounds such as amino acids (Fig. 1b). The generated reductive organic nitrogen compounds can be used as electron donors for the photoreduction of CO_2_. Meanwhile, *P. azotofixans* can create a local anaerobic environment, which allows the biohybrid system to continue photochemical CO_2_ reduction aerobically. This can reduce ROS generation and is important for practical application. Photoreduction studies of ^13^C-isotope-labeled CO_2_ demonstrate that the light-driven biohybrid system can perform photosynthesis of formic acid from CO_2_. Microbial metabolomics analysis combined with photocatalytic molecular mechanism studies reveals that the incorporated cobalt-based photocatalysts can utilize reduced organic nitrogen to mediate electron and proton transfers for the photoreduction of CO_2_. In particular, this biohybrid system shows a high apparent quantum efficiency of over 2.25% for photochemical CO_2_ reduction, and the organic nitrogen content undergoes an over-3-fold increase within 48 hours. This light-driven biohybrid system inherits both the high catalytic efficiency of artificial photocatalysts and the unique metabolic pathways of N_2_-fixing bacteria.

## RESULTS AND DISCUSSION

**Figure 2.**
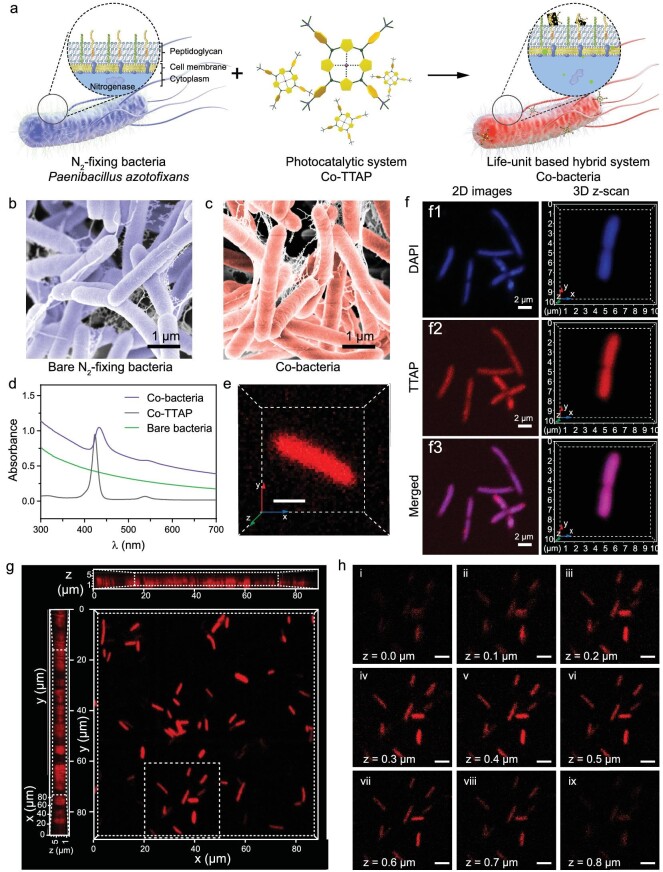
The construction and characterization of the light-driven biohybrid system. (a) Illustration of the construction of Co-bacteria. This biohybrid was constructed by incorporating molecular cobalt-based photocatalyst Co-TTAP into non-photosynthetic N_2_-fixing bacteria *P. azotofixans*. (b) SEM image of bare N_2_-fixing bacteria. (c) SEM image of Co-bacteria. (d) UV-vis absorbance spectra of free Co-TTAP, bare N_2_-fixing bacteria and Co-bacteria. (e) Confocal 3D image showing TTAP fluorescence distribution of different z scan planes in bacteria. Scale bar, 2 μm. (f) Confocal fluorescence images showing intracellular DNA and TTAP distribution. The DNA was stained with DAPI. On the left are confocal 2D fluorescence images. On the right are confocal 3D fluorescence images. (g) Fluorescence intensity analysis of Co-bacteria. (h) Confocal fluorescence images of Co-bacteria on different focal planes along the z direction.

In this work, we first prepared the light-driven biohybrid system containing N_2_-fixing bacterium *P. azotofixans* (ATCC 35681) with its biologically incorporated Cobalt(II) 5,10,15,20-tetra (4′-N, N, N-trimethylanilinium) porphyrin (Co-TTAP) photocatalyst (Fig. 2a). The structure of water-soluble TTAP was confirmed by 1H nuclear magnetic resonance (NMR) spectroscopy (Fig. S1). N_2_-fixing bacteria were inoculated and cultured in a nitrogen fixation medium (without N element) for 48 hours before the Co-TTAP solution was added (see Methods for details). In the following 2 hours, the N_2_-fixing bacteria were cultured in the presence of Co-TTAP. They were then centrifuged and separated from the supernatant. The obtained N_2_-fixing bacteria/Co-TTAP biohybrids (Co-bacteria) were re-dispersed in a nitrogen fixation medium. Scanning electron microscopy (SEM) was performed to characterize the morphology of the biohybrid system. It was found that the incorporated Co-TTAP had no obvious effect on the morphology of N_2_-fixing bacteria (Fig. 2b and c). The ultraviolet-visible (UV-vis) absorption spectrum of the re-dispersed Co-bacteria solution displayed the same characteristic peaks as the free Co-TTAP solution, compared to the featureless absorption peaks of the bare N_2_-fixing bacteria solution (Fig. 2d), indicating that the Co-TTAP remains intact during the incorporation process. After N_2_-fixing bacteria were co-cultured with Co-TTAP for 2 hours, Co-TTAP was preferentially taken up by N_2_-fixing bacteria with a 60.45% uptake efficiency (Figs S2 and S3). Each of the Co-bacterium contains ∼1.26 × 10^7^ Co-TTAP. Furthermore, the UV-vis absorption spectra of the re-dispersed solution of Co-bacteria showed no significant change within 24 hours, while the supernatant was nearly clear, suggesting that Co-TTAP could be well retained within N_2_-fixing bacteria (Fig. S4).

To investigate the distribution of molecular photocatalysts on N_2_-fixing bacterium, we performed a confocal 3D fluorescence z-scanning analysis of Co-bacteria and co-localization analysis of intracellular biomarkers and Co-TTAP. Super-resolution confocal microscopy was further utilized to clarify the location of molecular cobalt-based photocatalysts in the N_2_-fixing bacteria by collecting the fluorescence from TTAP under excitation at 630 nm. The confocal fluorescence images clearly showed that the molecular photocatalyst can be anchored in N_2_-fixing bacteria (Fig. 2e; Figs S5 and S6). Furthermore, a DNA-specific fluorescent probe, 4',6-diamidino-2-phenylindole (DAPI), was used to mark the DNA of bacteria. We found that the fluorescence of DAPI exhibited spatial overlap with the fluorescence of the molecular photocatalyst. This result indicated that the incorporated photocatalysts could be well dispersed across the whole N_2_-fixing bacterium (Fig. 2f, Fig. S7). Confocal 3D fluorescence z-scanning was also performed in order to analyze the distribution of photocatalysts in the N_2_-fixing bacteria. The images of several Co-bacteria on different fluorescence focal planes with a step increment of 100 nm along the z direction displayed the fluorescence intensity profile from the bottom to the top cross-sections of bacteria, suggesting that molecular photocatalysts could disperse across the whole bacterium (Fig. 2g and h).

**Figure 3.**
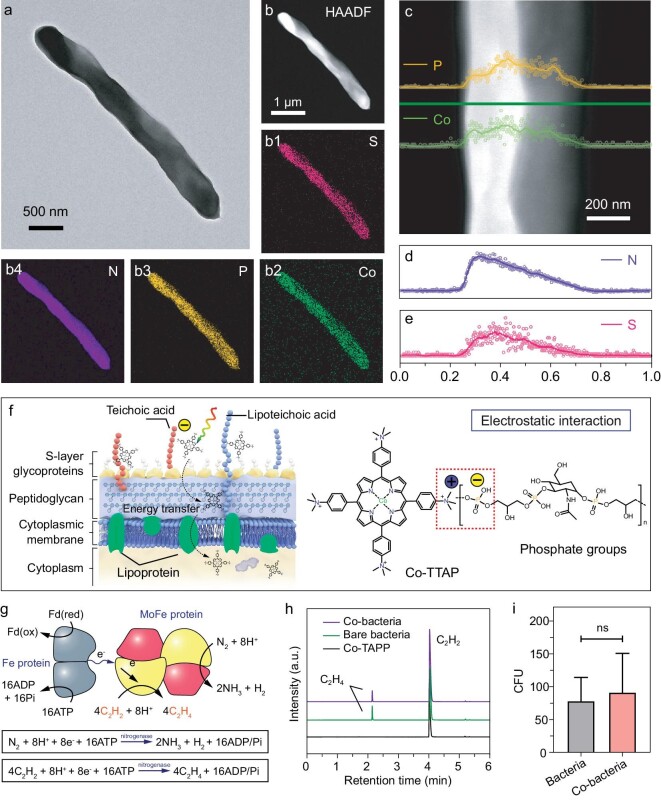
Analyses of the structure and bioactivity of Co-bacteria. (a) High-resolution transmission electron microscopy image of Co-bacteria. (b) High-angle annular dark field (HAADF)-STEM image of Co-bacteria. EDS mapping of the region in Fig. 3b showing the elements S (b1), Co (b2), P (b3) and N (b4) across the entire cell. (c–e) EDS line scanning of partially enlarged view of Fig. 3b and the distribution of elements P, Co, N (d) and S (e) along the green line. (f) Schematic diagram of the potential interactions between bacterium structures and Co-TTAP. (g) Illustration of the detection of nitrogenase activity via the catalytic reduction reaction of acetylene. (h) Gas chromatography analysis of the reaction product of Co-TTAP, bare N_2_-fixing bacteria and Co-bacteria, by using a flame ionization detector (FID) via the acetylene reduction assay. (i) Colony-forming unit assay of bare N_2_-fixing bacteria and Co-bacteria.

High-angle annular dark-field scanning transmission electron microscopy (HAADF-STEM) and energy-dispersive X-ray spectroscopy (EDS) mapping were carried out to verify the location of the cobalt-based photocatalyst on the surface of bacteria (Fig. 3a). As shown in Fig. 3b, the cobalt element can be evenly distributed across the entire bacterium. Meanwhile, Scanning transmission electron microscopy based energy-dispersive X-ray spectroscopy (STEM-EDS) line scanning was performed to analyze the element distribution on the bacterial surface (Fig. 3c–e, Fig. S8). It was found that the distribution curve of Co and that of P are almost similar, indicating that Co-TTAP has spatial overlap with biomolecules containing P on the surface of bacteria (Fig. 3c and f). This result indicates that Co-TTAP could be bound to the phosphoric groups on the bacterial wall through electrostatic interaction. The utilized N_2_-fixing bacterium *P. azotofixans* is Gram-positive. The bacterial surface is rich in teichoic acid containing abundant phosphoric groups, which leads to a relatively negative zeta potential on the bacterial surface [41,44]. Furthermore, zeta potential analysis was performed to monitor the surface potential change of N_2_-fixing bacteria, suggesting that incorporating Co-TTAP into N_2_-fixing bacteria can lead to a significant increase in the zeta potential of N_2_-fixing bacteria (Fig. S9). Molecular photocatalyst Co-TTAP has significantly positive charge, which could be bound to the phosphoric groups on the bacterial wall through electrostatic interaction, and can cross the bacterial membrane and then enter the bacteria (Fig. S10).

The viability of N_2_-fixing bacteria incorporating Co-TTAP was determined by counting colony-formed units (CFUs). The growth curve of N_2_-fixing bacteria co-cultured with Co-TTAP was obtained by diluting bacteria (Fig. S11). When the dilution time reached 10^5^, N_2_-fixing bacteria incorporating Co-TTAP showed no significant difference in the number of colonies compared to N_2_-fixing bacteria without Co-TTAP treatment, indicating that the viability and reproductive ability of N_2_-fixing bacteria were not affected by the incorporation of Co-TTAP (Fig. 3i). Meanwhile, the study of live/dead assays for Co-bacteria revealed that the viability of N_2_-fixing bacteria is not significantly affected by incubation with Co-TTAP (Figs S12 and S13). Furthermore, the nitrogenase activity of the Co-bacteria biohybrid system was measured by using the acetylene (C_2_H_2_) reduction method (Fig. 3g) [34,45]. The amounts of C_2_H_2_ and the produced ethylene (C_2_H_4_) were determined by gas chromatography (GC) (Fig. 3h, Figs S14–S18 and Tables S1–S3). The protein content of bacteria was obtained by the Bradford assay (Fig. S19). The result of nitrogenase activity analysis, consistent with total organic nitrogen content, revealed that the incorporation of Co-TTAP into N_2_-fixing bacteria has no effect on nitrogenase activity (Figs S20 and S21). These results indicated that Co-bacteria have high viability and can inherit the N_2_-fixation ability of bare N_2_-fixing bacteria.

**Figure 4.**
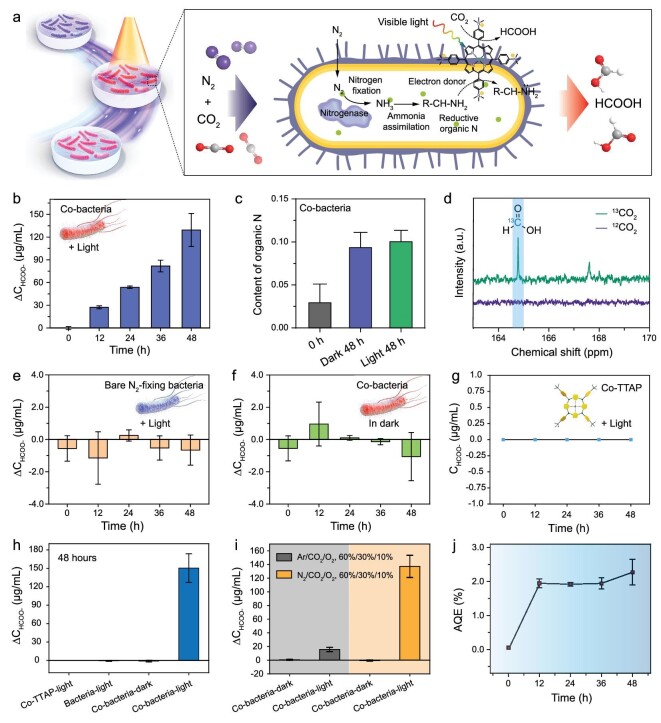
The performance of CO_2_ photoreduction and N_2_ fixation of Co-bacteria. (a) Schematic diagram showing that Co-bacteria enable the photosynthesis of formic acid from CO_2_ through N_2_ fixation and photocatalytic CO_2_ reduction. (b) The concentration of HCOO^−^ changed as light irradiation time for Co-bacteria. (c) Organic nitrogen content analysis of Co-bacteria under different treatment conditions. (d) ^13^C NMR spectrum of liquid product from ^13^CO_2_ and ^12^CO_2_ photoreduction by Co-bacteria for 24 hours under light irradiation. (e) The concentration of HCOO^−^ changed as light irradiation time increased for bare N_2_-fixing bacteria. (f) The concentration of HCOO^−^ changed as culture time increased in dark for Co-bacteria. (g) The concentration of HCOO^−^ changed as light irradiation time increased for Co-TTAP + nitrogen fixation medium group. (h) Amount of HCOO^−^ produced in different groups. (i) The photocatalytic tests of Co-bacteria were performed under different mixed-gas atmospheres. (j) AQEs of Co-bacteria changed as light irradiation time increased.

Photochemical CO_2_ reduction measurements were performed under visible light irradiation (λ ≥ 420 nm). The Co-bacteria were dispersed into a nitrogen fixation medium (without N) and then were transferred into a standard gas-liquid-solid reactor under a mixed-gas atmosphere that is composed of 60% N_2_, 30% CO_2_ and 10% O_2_ (Fig. S22). An illustration of the potential catalytic processes of Co-bacteria for N_2_ fixation and CO_2_ photoreduction is shown in Fig. 4a. The liquid products were detected and quantified by using ion chromatography along the photocatalytic process, showing a time-dependent increase for HCOO^−^ under continuous visible light illumination (Fig. 4b, Fig. S23). The gaseous products can be detected and quantified by GC after the catalytic cycle (Figs S24–S27). No gaseous products were detected by GC and gas chromatography-mass spectrometry (GC-MS), suggesting that Co-bacteria cannot convert CO_2_ into gaseous carbon products (Table S4). The average generation rate of HCOO^−^ for 48 hours was determined as (1.41 ± 0.25) × 10^−14^ mol h^−1^ cell^−1^. The organic nitrogen content underwent an over-3-fold increase over 48 hours (Fig. 4c). Meanwhile, the organic nitrogen content of Co-bacteria slightly increased compared to N_2_-fixing bacteria. This may be attributed to the fact that the incorporated Co-TTAP can improve the activity of nitrogenase under visible light irradiation [9]. Photocatalytic experiments were also performed using ^13^C-isotope-labeled CO_2_ to verify the origin of HCOO^−^, where ^13^C-isotope-labeled HCOO^−^ can be detected by ^13^C NMR spectroscopy. The ^13^C NMR spectrum clearly showed a peak at 164.8 ppm, corresponding to the ^13^C-isotope-labeled HCOO^−^ ion [46], when isotope ^13^C-isotope-labeled CO_2_ was utilized, while the signal was not obtained with ^12^CO_2_, excluding the contribution from other carbon species (Fig. 4d). The ^1^H NMR spectrum also confirmed the generation of HCOOH in the photocatalytic system with Co-bacteria (Fig. S28). These results suggested that Co-bacteria can convert CO_2_ into HCOO^−^ under visible light irradiation.

To investigate whether the innate metabolic pathways in N_2_-fixing bacteria can be used for CO_2_ reduction, we also carried out CO_2_ reduction measurements by utilizing bare N_2_-fixing bacteria as photocatalysts in a nitrogen fixation medium for CO_2_ reduction under similar conditions. No obvious increase in the amount of HCOO^−^ was observed by ion chromatography and no gaseous carbon products were detected by GC, indicating that bare N_2_-fixing bacteria cannot perform effective CO_2_ photoreduction reactions (Fig. 4e). Furthermore, when the CO_2_ reduction reactions of Co-bacteria were performed in the dark, the amount of HCOO^−^ showed no obvious change and no gaseous carbon species were detected, suggesting that Co-bacteria cannot carry out the catalytic synthesis of HCOO^−^ in dark conditions (Fig. 4f). These results suggest that although N_2_-fixing bacteria are capable of efficient N_2_ fixation, their unique metabolic pathways are not available for CO_2_ reduction.

To explore whether nitrogen fixation medium components such as glucose and mannitol, can be used as sacrificial electron donors for CO_2_ photoreduction, we performed CO_2_ photoreduction measurements in a nitrogen fixation medium by utilizing free Co-TTAP as a photocatalyst. No HCOO^−^ or gaseous carbon products were detected by ion chromatography and GC during the photocatalytic reaction, suggesting that Co-TTAP cannot utilize organics such as glucose and mannitol, in the nitrogen fixation medium, as electron donors for CO_2_ photoreduction (Fig. 4g). This could also rule out the possibility of a light-driven reaction, initiated by Co-TTAP, that degrades glucose and mannitol in the medium into formic acid. Notably, Co-bacteria can effectively produce HCOO^−^ under visible light irradiation compared with control groups such as bare N_2_-fixing bacteria and free Co-TTAP photocatalysts (Fig. 4h).

To verify the effect of N_2_ fixation on CO_2_ photoreduction, photocatalytic tests of Co-bacteria were performed in the absence of N_2_ under a mixed-gas atmosphere that consisted of 60% Ar, 30% CO_2_ and 10% O_2_. It was found that there was only a slight increase in the amount of HCOO^−^ in 48 hours (Fig. 4i). The photocatalytic tests of Co-bacteria were performed under different mixed-gas atmospheres, suggesting that N_2_ fixation is required for photocatalytic CO_2_ reduction in a biohybrid system of Co-bacteria (Fig. S29). Notably, while N_2_-fixing bacteria *P. azotofixans* needed to consume organic carbon sources for growth in this study, this bacterium can tolerate extreme environments and interact with many plants [42]. This provides the opportunity for the biohybrid system of Co-bacteria to utilize natural products to perform photocatalytic CO_2_ reduction.

The apparent quantum efficiency (AQE) of photochemical CO_2_ reduction was tested using visible light irradiation, and the AQE value at 48 hours was identified in Co-bacteria as 2.25 ± 0.38%, accounting for the number of excited electrons consumed to generate the product (Fig. 4j). The turnover number (TON) of CO_2_ photoreduction mediated by the biohybrid system for 24 hours was determined to be 9510. Furthermore, the turnover frequency (TOF) value of the photocatalyst in the biohybrid system was derived as 396.25 h^−1^. Additionally, the heterogeneous Co-bacteria can be easily separated and recovered from the reaction by centrifugation. After a photocatalytic test for 48 hours, the Co-bacteria were collected, centrifuged and re-dispersed in the culture medium. The morphology of Co-bacteria shows no obvious change in their SEM images, with relatively low magnification (Fig. S30). transmission electron microscopy elemental mapping displayed the element distribution within Co-bacteria, indicating that the composition of Co-bacteria also did not change significantly after CO_2_ photoreduction tests (Fig. S31). CFU assays were performed to evaluate the viability of Co-bacteria after photocatalytic testing for 48 hours, showing that N_2_-fixing bacteria can grow well (Figs S32 and S33). Meanwhile, the nitrogenase activity of Co-bacteria can still be well maintained after CO_2_ photoreduction (Fig. S34). These results indicate that N_2_-fixing bacteria still maintain good bioactivity after the photocatalytic reaction and have the potential to be recycled. After four cycles, each cycle being 24 hours in duration, the photocatalytic efficiency of Co-bacteria shows no significant decrease (Fig. S35).

**Figure 5.**
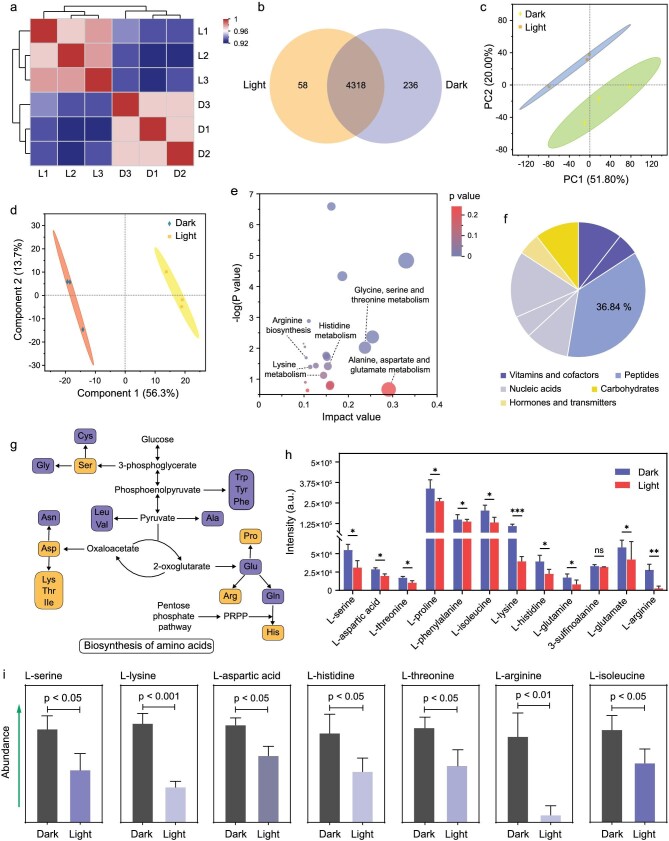
Metabolomics analysis of Co-bacteria showing dynamic changes of metabolite expression level. (a) Correlation analysis between Co-bacteria in the dark and Co-bacteria after photocatalytic reaction. (b) A Venn diagram showing the common and differential metabolites between dark and light groups. (c) Principal component analysis (PCA) of differential metabolites was used to assess the differences between dark and light groups. (d) Partial least square discriminant analysis (PLS-DA) of differential metabolites was used to assess the differences between dark and light groups. (e) KEGG topological analysis of different metabolites between dark and light groups. (f) KEGG compound classification analysis of different metabolites between dark and light groups. (g) Analysis of the KEGG functional pathway of biosynthesis of amino acids. The orange boxes indicate that the amino acid content has changed significantly. (h and i) Analysis of the contents of representative amino acids in metabolites.

To further reveal the fundamental reasons behind effective N_2_ fixation and photochemical CO_2_ reduction, we analyzed the metabolomics of Co-bacteria under visible light irradiation (light group) and in the dark (dark group). Both dark and light groups exhibited a high correlation coefficient, indicating that metabolomics has good biological reproducibility (Fig. 5a). Notably, there is a significant difference between dark and light groups for the correlation coefficient. In the metabolomics analysis, 4612 metabolites were detected and identified by inductively coupled plasma-mass spectrometry (ICP-MS). Among them, there are 4318 metabolites that are shared by dark group and light group, 236 metabolites that are unique to the dark group, and 58 metabolites that are unique to the light group (Fig. 5b). Principal component analysis (PCA) was performed to evaluate the differences between the dark and light groups, and showed that the differences for principal component 1 and principal component 2 reached 51.8% and 20.0%, respectively (Fig. 5c). The partial least square discriminant analysis (PLS-DA) score chart is used to visually display the classification effect of the model. As shown in Fig. 4d, principal component 1 was clearly separated by the PLS-DA model. The variation between groups reaches 56.3%. These results, combined with the orthogonal partial least squares discriminant analysis (OPLS-DA), suggest that the photochemical CO_2_ reduction mediated by Co-TTAP can significantly change the metabolites of N_2_-fixing bacteria (Fig. S36).

After the photocatalytic reaction, the differentially expressed metabolites were analyzed via the kyoto encyclopedia of genes and genomes (KEGG) database. These metabolites can be classified according to their participating metabolic pathways and performed functions. Functional pathway analysis shows that the top three pathways with the most changes in the number of metabolites are membrane transport, carbohydrate metabolism and amino acid metabolism (Fig. S37). Both KEGG topological analysis and KEGG pathway enrichment analysis for metabolites display many representative amino acid metabolic pathways, such as the glycine, serine, histidine and threonine metabolisms (Fig. 5e, Fig. S38). Furthermore, the metabolites with significant changes were classified through KEGG compound classification analysis. It was found that peptides accounted for the largest proportion, reaching 36.84% (Fig. 5f). Considering the significant changes of amino acids and peptides and the effect of N_2_ on CO_2_ photoreduction for Co-bacteria, we proposed that the incorporated Co-TTAP can utilize the reduced forms of nitrogen from N_2_-fixing bacteria as electron donors for photochemical CO_2_ reduction. To verify this, we analyzed the content changes of amino acids in the metabolomics of Co-bacteria. It was found that many amino acid metabolites, such as L-Serine, L-Aspartic acid, L-Threonine, L-Isoleucine, L-Lysine, L-Histidine and L-Arginine, significantly decreased after the photocatalytic reaction (Fig. 5g–i). These results indicate that N-containing organics, such as amino acids that are produced by N_2_-fixing bacteria, could be involved in the photocatalytic reaction. Although other reduced compounds (i.e. NAD(P)H) could be used as potential sacrificial donors, these compounds are limited and cannot be synthesized sustainably in Co-bacteria.

**Figure 6.**
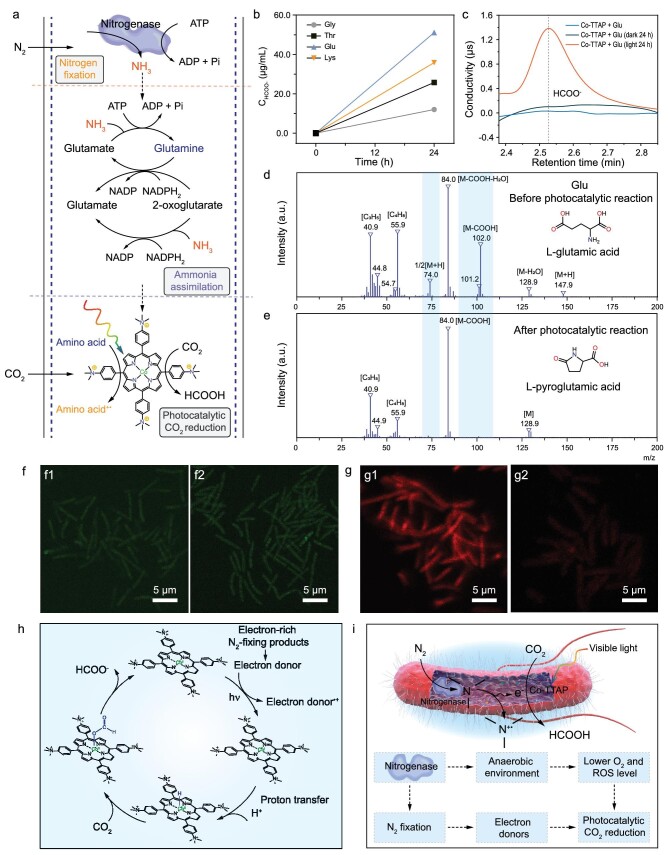
Mechanisms of Co-bacteria for N_2_ fixation and CO_2_ photoreduction. (a) Proposed mechanism of Co-bacteria for N_2_ fixation and photocatalytic CO_2_ reduction. (b) Photocatalytic CO_2_ reduction of Co-TTAP for the generation of HCOO^−^ by using different amino acids as sacrificial electron donors. (c) Ion chromatography analysis of the photocatalytic product over Co-TTAP using glutamic acid (Glu) as the sacrificial agent. (d) High-resolution mass spectrometry analysis of Glu. (e) High-resolution mass spectrometry analysis of Glu after photocatalytic reduction reaction. (f) Fluorometric intracellular ROS analysis of Co-bacteria. f1, Co-bacteria in the dark. f2, Co-bacteria under light irradiation. (g) Intracellular oxygen analysis of N_2_-fixing bacteria. g1, live N_2_-fixing bacteria; g2, dead N_2_-fixing bacteria. (h) Proposed mechanism of Co-TTAP for photocatalytic CO_2_ reduction. (i) Illustration of mechanisms for synergistic N_2_ fixation and photocatalytic CO_2_ reduction within Co-bacteria.

The proposed mechanism of Co-bacteria for N_2_ fixation and photocatalytic CO_2_ reduction is shown in Fig. 6a. N_2_-fixing bacteria *P. azotofixans* can convert atmospheric N_2_ into biologically available ammonia that can be utilized by transamination and ammonia assimilation processes for incorporation into nucleic acids and amino acids (Fig. 6a) [5,38,41]. Co-TTAP is a well-studied organic photocatalyst with an appropriate redox potential (Fig. S39) and is suitable for photochemical CO_2_ reduction (Fig. 6h) [47–49]. We demonstrated that triethanolamine (TEOA), a commonly used sacrificial electron donor, can mediate CO_2_ photoreduction of Co-TTAP to HCOO^−^ (Fig. S40). To confirm whether reduced forms of nitrogen from N_2_-fixing bacteria participate in photocatalytic CO_2_ reduction, we carried out CO_2_ photoreduction measurements of Co-TTAP by using various amino acids such as glycine (non-polar), tryptophan (neutral), glutamic acid (acidic) and lysine (alkaline), as external electron donors in aqueous solution. After photocatalytic reaction for 24 hours, the obvious generation of HCOO^−^ was observed by ion chromatography (Figs 6b, 5c and S41). No gaseous products were detected by GC (Fig. S42, Table S5). We monitored the structure change of amino acid in the photocatalytic reaction by GC-MS. Results of GC-MS clearly showed deprotonation, suggesting that amino acid could mediate electron and proton transfers for photochemical CO_2_ reduction (Fig. 6d and e). The reductively quenched process of molecular photocatalyst Co-TTAP was investigated by a steady-state fluorescence quenching experiment using glutamic acid as the quencher (Fig. S43). It was found that the quenching rate constant increases gradually with an increase in temperature, indicating that the process is dynamic (Fig. S44).

N_2_-fixing bacteria can rapidly consume oxygen gas for the respiration process, and thus maintain an anaerobic environment within the cell that can protect the nitrogenase from oxygen gas, without differentiating the cell or separating the N_2_ fixation from oxygen gas in different types of cells [36,41]. The anaerobic environment within the cells of N_2_-fixing bacteria could significantly inhibit the intracellular ROS generation that was induced by Co-TTAP. Meanwhile, *P. azotofixans* can also express superoxide dismutase to reduce intracellular oxidative stress [34,39]. Therefore, we used an ROS assay kit to detect the ROS level within the cytoplasm of N_2_-fixing bacteria by recording the ROS-triggered fluorescence emission intensity. Compared to bare N_2_-fixing bacteria, there was no significant increase in the ROS concentration for Co-bacteria under visible light irradiation (Fig. 6f). Furthermore, we applied the sensing probe to assay the O_2_ level in the cytoplasm of N_2_-fixing bacteria. It was found that the hypoxic fluorescence of Co-bacteria is significantly stronger than that of dead N_2_-fixing bacteria, indicating that the O_2_ concentration in Co-bacteria is much lower than in the dead N_2_-fixing bacteria, where the air can enter freely (Fig. 6g, Figs S45 and S46). The ability to facilitate aerobic N_2_ fixation and photochemical CO_2_ reduction is an especially novel aspect of this light-driven biohybrid system that enables applications under milder and more environmentally benign conditions (Fig. 6i). In addition, we investigated the effects of different pH and temperatures on the viability of the biohybrid system. The suitable pH and temperature for the growth of N_2_-fixing bacterium *P. azotofixans* are 7.2 and 30°C, respectively. It was found that the cell viability of the biohybrid system was significantly inhibited at pH 4.0, 5.5 and 10.0 (Fig. S47). Notably, the cell viability of the biohybrid system showed a slight decrease at 4, 15 and 37°C (Fig. S48).

## CONCLUSION

In summary, we have demonstrated that Co-TTAP, acting as an intercellular photocatalyst, could enable non-photosynthetic N_2_-fixing bacteria *P. azotofixans* to achieve efficient photochemical reduction of CO_2_. A molecular organic cobalt-based photocatalyst with high biocompatibility inside the N_2_-fixing bacteria enables the photosynthesis of formic acid from CO_2_ without using external electron donors. Furthermore, we found that N_2_-fixing bacteria can utilize N_2_ to produce reduced organic nitrogen such as amino acids and dangling amino acid residues, as electron donors for the photoreduction of CO_2_. This biohybrid system eradicates the need for external electron donors in photosynthetic biohybrid systems previously reported (Table S6). The biohybrid system showed a high AQE of over 2.25% for photochemical CO_2_ reduction under visible light irradiation. More importantly, the organic nitrogen content underwent an over-3-fold increase within 48 hours. Besides the high photocatalytic efficiency and the increase of organic nitrogen content, continuous CO_2_ photoreduction over 4 days benefited from the high viability of the Co-bacteria biohybrid system, with a local anaerobic environment in the N_2_-fixing bacteria. This work realized the coupling of CO_2_ conversion and N_2_ fixation under ambient conditions, providing a promising platform for utilizing the abundant N_2_ in the air to achieve effective photochemical CO_2_ reduction, which is important for sustainable development.

## METHODS

The supplementary data includes the experimental details, molecular structure analysis, UV-Vis absorbance spectra analysis, uptake efficiency analysis, stability analysis, confocal fluorescence analysis, TEM-EDS analysis, colony forming unit assay, gas chromatography analysis, nitrogenase activity analysis, total organic nitrogen content analysis, intracellular oxygen analysis and microbial metabolomics analysis.

## Supplementary Material

nwad142_Supplemental_File
