# Supplementary material for: Light-driven biohybrid system utilizes N2 for photochemical CO2 reduction
Source: Natl Sci Rev. 2023 May 15;10(7):nwad142. doi: 10.1093/nsr/nwad142 (PMC10325001; doi:10.1093/nsr/nwad142)
Supplement: nwad142_Supplemental_File [file nwad142_supplemental_file.pdf]

Supplementary data

of

Light-driven biohybrid system utilizes N_2 for photochemical CO_2 reduction

Jin-Yue Zeng[1,†], Xiao-Shuang Wang[1,†], Xin-Hua Liu[1], Qian-Ru Li[1], Jun Feng[1] and Xian-Zheng Zhang[1,2,*]

[1]Key Laboratory of Biomedical Polymers of Ministry of Education & Department of Chemistry, Wuhan University, Wuhan 430072, China;

[2]Wuhan Research Centre for Infectious Diseases and Cancer, Chinese Academy of Medical Sciences Wuhan 430071, China

***Corresponding author.** E-mail: xz-zhang@whu.edu.cn

[†]Equally contributed to this work.

Section 1 Synthesis and characterizations

Materials. NaCl, $CaCl_2 \cdot 2H_2O$, $MgSO_4 \cdot 7H_2O$, $Na_2MoO_4 \cdot 2H_2O$, K_2HPO_4, KH_2PO_4, $FeSO_4 \cdot 7H_2O$, and $CaCO_3$, Glucose and mannitol were purchased from Sinopharm Chemical Reagent Co., Ltd. Tryptone and soy peptone were obtained from Coolaber Science & Technology. All chemicals were analytical grade and used as received. Agar and Bradford protein assay kit were purchased from Beyotime Biotechnology.

Characterizations. Scanning electron microscopy (SEM) images were taken using a field emission scanning electron microscope (Zeiss SIGMA). Transmission electron microscope (TEM) images were obtained on a JEM-F200 transmission electron microscope. The UV-Vis spectra were obtained with a Lambda 35 (Perkin Elmer) spectrophotometer. Zeta potential was tested by Nano-ZS ZEN3600 (Malvern Instruments). Gas chromatography was performed on Fast refinery gas analysis system based on the Agilent7890B GC system. Ion chromatography was performed on an ion chromatography (DIONEX ICS 2500). Confocal laser scanning microscopy (CLSM) images were collected on a laser scanning confocal microscope (Leica TCS SP8 STED). The ^{1}H and ^{13}C Nuclear Magnetic Resonance (NMR) spectra were recorded on a Bruker Avance III HD 400 MHz.

Synthesis of TTAP. 4,4',4'',4'''-(porphyrin-5,10,15,20-tetrayl)tetraaniline (100.0 mg, 0.15 mmol) was dispersed into 20.0 mL of DMF. The mixed solution was added to a 50.0 mL flask after dissolved ultrasonically. Then, the flask was sealed and heated to 40 ℃. 1.0 mL of methyl iodide was injected into the flask quickly. After 48 h, a large amount of precipitation appeared at the bottom of the flask. The reaction solution was transferred to a centrifuge tube and precipitation was collected with centrifugation (8000 rpm, 5 min), followed by washing twice with ethanol. The resulting product was washed with ethanol for three times and dried in vacuum at 60 ℃ to give 115.0

mg of TTAP.

Synthesis of Co-TTAP. 100.0 mg TTAP was added into 100.0 mL of water, stirred and heated to 40 ℃. When TTAP was dissolved, the solution was cooled down and its pH was adjusted to 7.0. After that, 250.0 mg $Co(Ac)_2 \cdot 4H_2O$ and 125.0 mg KI were added. The mixed solution was bubbled with N_2 and kept into a water bath at 55 ℃ overnight under N_2 atmosphere. Finally, the solution was cooled down and concentrated under reduced pressure to 50.0 mL. Precipitation formed at the bottom of the flask and collected with centrifugation (8000 rpm, 5 min), followed by washing with acetone and water sequentially. The resulting product was dried in vacuum at 60 ℃ to give 80.0 mg of Co-TTAP.

Preparation of nitrogen fixation medium. To prepare the nitrogen fixation medium (without nitrogen), 5.0 g of glucose, 5.0 g of mannitol, 100.0 mg of $CaCl_2 \cdot 2H_2O$, 100.0 mg of $MgSO_4 \cdot 7H_2O$, 5.0 mg of $Na_2MoO_4 \cdot 2H_2O$, 0.9 g of K_2HPO_4, 0.1 g of KH_2PO_4, 10.0 mg of $FeSO_4 \cdot 7H_2O$, 5.0 g of $CaCO_3$ were added into 1.0 L of deionized water and stirred until dissolved. The pH of nitrogen fixation medium is about 7.2. The nitrogen fixation medium was autoclaved at 121 ℃ for 30 min. When the temperature dropped to 60 ℃, the medium was taken out. The medium was quickly poured into the prepared sterile petri dish. The petri dish was gently shaken to distribute the medium evenly and then placed on the bench. All operations were carried out under a sterile environment.

Preparation of heterotrophic medium. To obtain the heterotrophic medium, 17.0 g of tryptone, 5.0 g of soy peptone, 5.0 g of NaCl, 2.5 g of glucose, and 2.5 g of K_2HPO_4 were added into 1.0 L of deionized water. After being stirred and fully dissolved, the medium was autoclaved for 30 min at 121 ℃. All operations were carried out under a sterile environment.

Strain identification. *Paenibacillus azotofixans* (*P. azotofixans*, ATCC 35681) is Gram-positive, facultative anaerobic, rod-shaped, and endospore-forming bacteria. The widely distributed *P. azotofixans* could tolerate extreme environments and interact with a variety of plants. After the N_2-fixing bacteria *P. azotofixans* were recovered and purified, their 16S rDNA were extracted for strain identification. The sequence is as follows:

AATCCCTCCGTGGTACCGTCCTCCCGAGGGTTAGACTAGCTACTTCTGGAG
CAACCCACTCCCATGGTGTGACGGGCGGTGTGTACAAGGCCCGGGAACGT
ATTCACCGCGACATTCTGATTCGCGATTACTAGCGATTCCGACTTCACGCAG
TCGAGTTGCAGACTGCGATCCGGACTACGATCGGTTTTCTGGGATTAGCTC
CGCCTCGCGACTTGGCAACCCTTTGTACCGACCATTGTAGCACGTGTGTAG
CCCTGGCCGTAAGGGCCATGATGACTTGACGTCATCCCCACCTTCCTCCGG
TTTGTCACCGGCAGTCTCCTTAGAGTGCCCGACCGAATCGCTGGTAACTAA
GGACAAGGGTTGCGCTCGTTACGGGACTTAACCCAACATCTCACGACACG
AGCTGACGACAGCCATGCAGCACCTGTCTCTGCGCTCCCGAAGGCACCCAT
CAATCTCTTGAAAGTTCGCAGGATGTCAAGGCCAGGTAAGGTTCTTCGCGT
TGCTTCGAATTAAACCACATGCTCCACCGCTTGTGCGGGCCCCCGTCAATT
CATTTGAGTTTTAACCTTGCGGCCGTACTCCCCAGGCGGTCGACTTAATGCG
TTAGCTGCGCCACTAAGCTCTCAAGGAGCCCAACGGCTAGTCGACATCGTT
TACGGCGTGGACTACCAGGGTATCTAATCCTGTTTGCTCCCCACGCTTTCGC
ACCTCAGTGTCAGTATCGGTCCAGGTGGTCGCCTTCGCCACTGGTGTTCCT
TCCTATATCTACGCATTTCACCGCTACACAGGAAATTCCACCACCCTCTACC
GTACTCTAGCCCAGTAGTTTTGGATGCGGTTCCCAGGTTGAGCCCGGGGCT
TTCACATCCAACTTGCCGAACCACCTACGCGCGCTTTACGCCCAGTAATTCC
GATTAACGCTTGCACCCTTCGTATTACCGCGGCTGCTGGCACGAAGTTAGC

CGGTGCTTATTCTGTCGGTAACGTCAAGACGCTTGGGTATTCACCGAGCGC

CCTTCCTCCCAACTTAAAGTGCTTTACAATCCGAAGACCTTCTTCACACAC

GCGGCATGGCTGGATCAGGCTTTCGCCCATTGTCCAATATTCCCCACTGCTG

CCTCCCGTAGGAGTCTGGACCGTGTCTCAGTTCCAGTGTGACTGATCATCC

TCTCAGACCAGTTACGGATCGTCGCCTTGGTGGGCCTTTACCCCACCAACT

AGCTAATCCGACCTAGGCTCATCTGTTAGCGTGAGGCCCGAGGGTCCCCCA

CTTTCTCCCGTAGGACGTATGCGGTATTAGCGCGAGTTTCCCCGCGTTATCC

CCCACTAACAGGCAGATTCCTAGGCATTACTCACCCGTCCGCCGCTCGCCG

GCAACCCGAAGGTCCCGCTGCCGCTCGACTTGCATGTGTAGCCCG

The 16S rDNA sequence is aligned on the gene database: Ribosomal Database Project (RDP) http://rdp.cme.msu.edu/classifier/classifier.jsp. Through the comparison, we determined the classification information of the test rDNA gene sequence from the phyla to the genus/species level and the corresponding confidence level for each level are given. The analyzed results were showed as following:

»rootrank Root (1 sequences) 100%

» »domain Bacteria 100%

» » »phylum Firmicutes 100%

» » » »class Bacilli 100%

» » » » »order Bacillales100%

» » » » » »family Paenibacillaceae 1 100%

» » » » » » »genus Paenibacillus 100%.

Section 2 Supplementary methods

Cultivation and inoculation of N_2-fixing bacteria. In this work, the used N_2-fixing bacterium was *Paenibacillus azotofixans* (formerly *Bacillus azotofixans*, ATCC 35681), which can be cryopreserved in a -80 ℃ freezer with 25% glycerin as a cryoprotectant. To obtain a large number of N_2-fixing bacteria, *Paenibacillus azotofixans* was initially cultured in the heterotrophic medium. Specifically, 500.0 μL of thawed cryopreserved N_2-fixing bacteria stock was added into 25.0 mL of heterotrophic medium, and incubated in a shaker (200 rpm) at 30 ℃. After the growth for 2 days, the N_2-fixing bacteria were collected by centrifugation at 6000 rpm for 5 min. The resulting bacteria were washed with PBS for three times, and re-suspended in 2.0 mL of PBS to obtain N_2-fixing bacteria stock solution. 100.0 μL of the bacteria stock solution was dispersed into 15.0 mL of nitrogen fixation medium. The N_2-fixing bacteria were cultured in nitrogen fixation medium at 30 ℃ for another 3 days. All operations were carried out under a sterile environment.

Incorporation of Co-TTAP into N_2-fixing bacteria. 100.0 μL of the bacteria stock solution was dispersed into 15.0 mL of nitrogen fixation medium. The N_2-fixing bacteria were cultured in nitrogen fixation medium at 30 °C for 3 days. 500.0 μL of nitrogen fixation medium solution containing Co-TTAP (20 μg/mL) was added to the plate, and co-cultured with N_2-fixing bacteria for 2 hours. The N_2-fixing bacteria incorporating Co-TTAP were collected by centrifugation at 6000 rpm for 5 min, and re-dispersed into nitrogen fixation medium for photocatalytic testing. Each N_2-fixing bacterium consists of approximately 1.26×10^7 of Co-TAPP molecules.

Laser scanning confocal microscope. The fluorescence imaging of biohybrid system was performed with confocal microscope (Leica TCS SP8 STED), equipped with air objectives. The N_2-fixing bacteria were cultured in nitrogen fixation medium at 30 ℃

for 3 days. 500.0 μL of nitrogen fixation medium solution containing photosensitizer (20 μg/mL) was added to the plate, and co-cultured with N_2-fixing bacteria for 2 hours. The N_2-fixing bacteria incorporating photosensitizer were collected by centrifugation at 6000 rpm for 5 min, and re-dispersed into 500 μL of PBS. 5 μL of bacteria suspension was dropped onto a clean glass slide, then a cover glass was placed on the drop. The bacteria were observed via a laser scanning confocal microscope.

Scanning electron microscopy (SEM). Bare N_2-fixing bacteria and Co-bacteria hybrid were collected and purified by centrifugation. Then, bare N_2-fixing bacteria and Co-bacteria were fixed by soaking in glutaraldehyde solution (2.5 vol.%) at 4 ℃ overnight. After centrifugation, bare N_2-fixing bacteria and Co-bacteria were subjected to ethanol dehydration by placing them in 35, 50, 70, 95 and 100% ethanol in PBS for 10 min each. The 100% ethanol was changed three times, and the samples were dried with N_2 purge. Finally, free N_2-fixing bacteria and Co-bacteria were adhered to SEM posts with carbon film tape and imaged with a SEM at 5 kV (Sigma).

Transmission electron microscopy (TEM). After the N_2-fixing bacteria were co-cultured with the Co-TTAP (20 μg/mL) for 4 hours, the obtained Co-bacteria were fixed with glutaraldehyde solution (2.5 vol.%) at 4 ℃ overnight. The fixed Co-bacteria were collected by centrifugation and re-dispersed into PBS. Samples of fixed Co-bacteria were prepared for TEM and EDS mapping by dropping the Co-bacteria suspension onto Cu TEM grids and settling for 2 hours. After the samples were washed briefly in deionized water, the grids were dried in air overnight. TEM imaging and EDS mapping was performed with a JEM-F200 transmission electron microscope at 200 kV.

Colony forming unit assay. The optical density of N_2-fixing bacteria suspension at 600 nm was adjusted to 0.3 with PBS. Then the above-mentioned bacteria suspension

was diluted 10^4, 10^5 and 10^6 times respectively to prepare the bacteria dilutions. 100 μL of the bacteria dilution was added to the plate loaded with nitrogen fixation medium. 500.0 μL of medium solution containing Co-TTAP (20 μg/mL) was added to the medium. The plate was sealed with parafilm, placed upside down and incubated in an incubator at 30 ℃ for 3 days. After the growth for 3 days, the white circular colonies were counted to determine the CFU mL^{-1} as a measure of bacteria number and viability. All operations were carried out under a sterile environment.

Determination of nitrogenase activity. The acetylene (C_2H_2) method was used to determine the nitrogenase activity of N_2-fixing bacteria. Briefly, 40.0 mL of nitrogen fixation medium was added to the glass bottle (100 mL). N_2-fixing bacteria were inoculated in nitrogen fixation medium and cultured at 30 ℃ for 3 days. 1.0 mL of Co-TTAP (20 μg/mL) medium solution was added to the medium containing N_2-fixing bacteria. After co-cultivation for 2 hours, N_2-fixing bacteria incorporating Co-TTAP were collected by centrifugation at 6000 rpm for 5 min. The obtained N_2-fixing bacteria containing Co-TTAP (Co-bacteria) were re-dispersed into 40.0 mL of nitrogen fixation medium in 100 mL-glass bottle. Control groups included Co-TAPP (containing the equal amount of Co-TAPP as Co-bacteria solution) and bare N_2-fixing bacteria. The glass bottles were sealed with rubber stoppers, and then 10.0 mL of acetylene was injected. After the growth for 3 days, the gas phase composition was analyzed by gas chromatography (Fast refinery gas analysis system based on the Agilent7890B GC system). The bacteria were collected for concentration (6000 rpm, 5 min). The protein content of bacteria was measured according to Bradford with bovine serum albumin as the standard. The activity of nitrogenase is calculated according to the following equation (1):

$$Nitrogenase\ activity = \frac{n_{C_2H_4}}{m*T} \quad (1)$$

Here, m refers to the quality of bacterial protein and T is the reaction time.

The nitrogenase activity determination of Co-bacteria after photocatalytic CO_2 reduction tests. The acetylene (C_2H_2) method was used to determine the nitrogenase activity. The Co-bacteria were re-dispersed into 40.0 mL of nitrogen fixation medium in 100 mL-glass bottle. Control groups included Co-TTAP and light-irradiated bare N_2-fixing bacteria. The glass bottles were sealed with rubber stoppers, and then 10.0 mL of acetylene was injected. After the growth for 3 days, the gas phase composition was analyzed by gas chromatography (Fast refinery gas analysis system based on the Agilent7890B GC system). The bacteria were collected for concentration (6000 rpm, 5 min). The protein content of bacteria was measured according to Bradford with bovine serum albumin as the standard. The activity of nitrogenase is calculated according to the following equation (1).

Photocatalytic CO_2 reduction tests. Prior to photocatalytic experiments, (2.08 ± 0.09) $\times 10^8$ of Co-bacteria (containing ~1.26 $\times 10^7$ Co-TAPP/cell) were inoculated in nitrogen fixation medium on the plate with a diameter of 4 cm. The plate was transferred into a standard gas-liquid-solid photo-reactor. Then, the photo-reactor was purged with a mixture of gases ($N_2/CO_2/O_2$, 6:3:1). The reactor was illuminated with a 300 W Xe lamp with 420-cut filter (removing light with wavelengths less than 420 nm) to obtain visible light (light intensity, 7.5 mW cm^{-2}). Liquid phase solution was centrifuged to remove Co-bacteria. The liquid products of photocatalytic process were analyzed by an ion chromatography (the sample is diluted 100 times). The composition of the gas atmosphere was monitored by gas chromatography (Fast refinery gas analysis system based on the Agilent7890B GC system). A three channel system on the Agilent7980B GC system was used for the determination of the composition of gas atmosphere in this photocatalytic system. Five valves and eight

columns are used with the system. Channel 1, using a Flame Ionization Detector (FID1), was used for hydrocarbons from methane to C6+. Permanent gases (i.e. O_2, N_2, CO_2 and CO) and hydrogen sulfide were measured on Channel 2, using a Thermal Conductivity Detector (TCD2). Hydrogen (H_2) was measured on the Channel 3, using a Thermal Conductivity Detector (TCD3), where N_2 was used for carrier. Control groups included: (1) free Co-TAPP in nitrogen fixation medium + light, (2) bare N_2-fixing bacteria + light, and (3) Co-bacteria in dark. Each group includes 3 parallel samples.

Trace the C source using $^{13}CO_2$. In order to trace the source of C in photocatalytic products, we performed photocatalytic reactions of Co-bacteria by using a mixture of gases containing $^{13}CO_2$ (N_2/$^{13}CO_2$/O_2, 6:3:1). Except that the $^{12}CO_2$ was replaced with $^{13}CO_2$, H_2O was replaced with D_2O, and the other photocatalytic treatments were the same as before. After 24 h, the reaction products were collected and analyzed by carbon spectroscopy on a Bruker Avance III HD 400 MHz.

Gas chromatography analysis. The composition of the gas atmosphere was monitored by gas chromatography (Fast refinery gas analysis system based on the Agilent7890B GC system). A three channel system on the Agilent7980B GC system was used for the determination of the composition of gas atmosphere in this photocatalytic system. Five valves and eight columns are used with the system. Channel 1, using a Flame Ionization Detector (FID1), was used for hydrocarbons from methane to C6+. Permanent gases (i.e. O_2, N_2, CO_2 and CO) and hydrogen sulfide were measured on Channel 2, using a Thermal Conductivity Detector (TCD2). Hydrogen (H_2) was measured on the Channel 3, using a Thermal Conductivity Detector (TCD3), where N_2 was used for carrier.

Materials and reagents of full analysis: carrier gas, N_2 (purity > 99.999%), H_2

(purity > 99.995%); fuel gas, H_2 (purity > 99.995%); oxidant gas, air; Auxiliary gas (septal purge and tail purge), N_2 (purity > 99.995%). Instrument conditions of full analysis: injection volume: FID1, 250 μL; TCD2, 100 μL; TCD3, 100 μL. Injection port: inlet temperature, 250 ℃; pressure, 9.9949 psi; total flow, 145 mL/min; septum purge, 3 mL/min. Split injection: split ratio, 70 : 1; shunt flow, 140 mL/min. Columns:

Column#1: 2 Ft Unibeads IS 60/80 mesh in UltiMetal;

Column#2: 4 Ft Unibeads IS 60/80 mesh in UltiMetal;

Column#3: 8 Ft Molecular Sieve 5A 60/80 mesh in UltiMetal;

Column#4: 3 Ft HayeSep Q 80/100 mesh in UltiMetal;

Column#5: 8 Ft Molecular Sieve 5A 60/80 mesh in UltiMetal;

Column#6: 123-1015(cut) 2 m × 0.32 mm × 5 um DB-1;

Column#7: 19091P-S12 25 m × 0.32 mm × 8 um HP-AL/S;

Column#8: 123-1015(cut) 0.45 m × 0.32 mm × 5 um DB-1.

Oven: program heating (60 ℃ for 1 minute; heating 20 ℃/min to 80 ℃; then heating 25 ℃/min to 190 ℃; keeping at 190 ℃ for 0.3 minutes).

Detector: FID1 (Heater, 250 ℃; Air flow, 350 mL/min; Hydrogen flow rate, 40.0 mL/min; Tail blowing flow rate (N_2), 27.0 mL/min), TCD2 (Heater, 250 ℃; Reference flow rate, 45.0 mL/min; Tail blowing flow rate, 2.0 mL/min) and TCD3 (Heater, 250℃; Reference flow rate, 45.0 mL/min; Tail blowing flow rate, 2.0 mL/min).

Photocatalytic CO_2 reduction tests of Co-TAPP. We investigated the photocatalytic activity of Co-TAPP by using different amino acids as sacrificial electron donors under full spectrum irradiation (λ ≥ 420 nm). Co-TAPP was dispersed in deionized water for photocatalytic measurements. A standard gas-liquid-solid reactor was used for the assessment of photocatalytic CO_2 reduction performance. In a typical

procedure, photocatalysts were dispersed in deionized water containing amino acid (50 mM) solution. The concentration of Co-TAPP photocatalyst was determined to 6.0 μg/mL (containing the equal amount of Co-TAPP as Co-bacteria solution). The mixture was pre-degassed with CO_2 for removing the dissolved O_2, and then injected into the gas-liquid-solid reactor. The reactor was illuminated using a 300 W Xe lamp (Purchased from Beijing China Education Au Light Technology Co., Ltd.) with UVIRCUT420 nm filter to obtain full spectrum irradiation light source (light intensity, 7.5 mW cm^{-2}). The irradiation time is 24 hours. The composition of the gas atmosphere was monitored by gas chromatography (Fast refinery gas analysis system based on the Agilent7890B GC system). The liquid products of photocatalytic process were analyzed by an ion chromatography (DIONEX ICS 2500).

Total organic nitrogen content analysis. $(2.08 \pm 0.09) \times 10^8$ of Co-bacteria (containing ~1.26×10^7 Co-TAPP/cell) were inoculated in nitrogen fixation medium (without N) on the plate. The plate was transferred into a standard gas-liquid-solid photo-reactor. Then, the photo-reactor was purged with a mixture of gases ($N_2/CO_2/O_2$, 6:3:1). The reactor was illuminated with a 300 W Xe lamp with 420-cut filter to obtain visible light (light intensity, 7.5 mW cm^{-2}). After the samples were irradiated for 48 hours, the medium containing bacteria and in the plate were freeze-dried for total organic nitrogen analysis, and the mass of the freeze-dried mixture was determined to be 245.8 ± 17.4 mg. Analyzes of total organic nitrogen content were performed on a FOSS automatic kjeldahl nitrogen analyzer. Control groups included: (1) free Co-TAPP in nitrogen fixation medium + light, (2) bare N_2-fixing bacteria + light, and (3) Co-bacteria in dark. Each group includes 3 parallel samples. Before irradiation, the culture medium containing Co-bacteria was freeze-dried for total organic nitrogen content analysis. The mass of the freeze-dried mixture was

determined to be 238.6 $\pm$11.2 mg.

Total organic nitrogen content analysis without light irradiation. (2.08 $\pm$0.09) $\times 10^8$ of Co-bacteria (containing ~1.26 $\times 10^7$ Co-TAPP/cell) were inoculated in nitrogen fixation medium (without N) on the plate. After the sample was cultured for 48 hours, the mixture containing bacteria and medium in the plate was collected for total organic nitrogen analysis. Analyzes of total organic nitrogen content were performed on a FOSS automatic kjeldahl nitrogen analyzer. Control groups included: (1) free Co-TAPP in nitrogen fixation medium, and (2) bare N_2-fixing bacteria. Each group includes 3 parallel samples.

Apparent quantum efficiency (AQE) study. The AQE was measured in identical experimental setup and under the same condition to the CO_2 photo-reduction test except for the incident light resource. Co-bacteria (containing ~1.26 $\times$ 10^7 Co-TAPP/cell) were inoculated in nitrogen fixation medium on the plates. The photo-reactor was purged with a mixture of gases (N_2/CO_2/O_2, 6:3:1). The typical irradiation area of photocatalytic system was 12.56 cm^2. The distance between photocatalytic solution surface and the light resource fixed at 10.0 cm throughout the entire catalytic experiment. The intensity of visible light was calibrated to be 7.5 mW cm^{-2} (averaged wavelength, 546 nm). The sample was irradiated for 48 hours and the photo-catalytic products were quantitatively detected by ion chromatography. The AQE value was calculated as the number of electrons consumed to generate the product divided by the total number of incident photos (Equation 2). This method has been widely adopted in the field of CO_2 photoreduction. Here, 2 electrons were used to convert one CO_2 molecule to HCOOH (Equation 3). Each AQE measurement was repeated at least three times in parallel to obtain a reproducible and reliable value with the error bar displayed.

$$AQE = \frac{The\, total\, number\, of\, consumed\, electrons}{The\, total\, number\, of\, incident\, photos} \times\ 100\% \ (2)$$

$$CO_2 + 2H^+ + 2e^- \rightarrow HCOOH \ (3)$$

Turnover number (TON) study. The turnover number (TON) is the number of reductions that occur per catalyst over the catalyst's lifetime. This is often calculated as the molar ratio of CO_2 reduction products to the catalyst initially present (Equation 4).

$$TON = \frac{Mole\, of\, formic\, acid\, generation}{Mole\, of\, photocatalyst} \ (4)$$

Turnover Frequency (TOF) Study. TOF is defined as the TON per unit of time (Equation 5).

$$TOF = \frac{Mole\, of\, formic\, acid\, generation}{(Mole\, of\, photocatalyst \times reaction\, time)} \ (5)$$

Microbial metabonomics study. (2.08 ± 0.09) $\times 10^8$ of Co-bacteria (containing ~1.26 $\times 10^7$ Co-TAPP/cell) were inoculated in nitrogen fixation medium on the plates. These plates were randomly divided into dark group and light group (each group includes 3 parallel samples). The plate was transferred into a standard gas-liquid-solid photo-reactor. Then, the photo-reactor was purged with a mixture of gases (N_2/CO_2/O_2, 6:3:1). The light group was illuminated with a 300 W Xe lamp with 420-cut filter (light intensity, 7.5 mW cm^{-2}). The control group (dark group) was placed in a dark environment. After treating for 24 hours, Co-bacteria were collected and preserved at -80 ℃.

50.0 mg of Co-bacteria for each sample was accurately weighed into a 2-mL centrifugation tube. Then, a grinding bead with a diameter of 6 mm and 400 μL of extraction solution containing the internal standard of L-2-Chlorophenylalanine (0.02 mg/mL) were added to the centrifuge tube. The frozen tissue grinder was used for grinding samples at a frequency of 50 Hz at -10 ℃. The sample was put into the

low-temperature ultrasound (40 kHz, 5 ℃) for 30 min to extract metabolites fully, and then placed at -20 ℃ for 30 min. After the sample is centrifuged, the supernatant is transferred to an injection vial with an inner cannula for analysis on the UHPLC-Q Exactive HF-X system (Thermo Fisher). Chromatographic conditions: chromatographic column is ACQUITY UPLC HSS T3 (100 mm × 2.1 mm i.d., 1.8 µm; Waters, Milford, USA). Mobile phase A is 95% water and acetonitrile (containing 0.1% formic acid). Mobile phase B is 47.5% acetonitrile, 47.5% isopropanol and 5% water (containing 0.1% formic acid). The injection volume is 2.0 µL, and the column temperature is 40 ℃. Metabolomics data were processed and analyzed by ProgenesisQI software. The metabolites were identified using standard databases such as the Human Metabolome Database (HMDB) and the METLIN database.

Electrochemical analysis. Cyclic voltammetry measurements were carried out on a CHI 760E electrochemical workstation in a conventional three electrode cell, using glassy carbon as the working electrode, Ag/AgCl as the reference electrode and the Pt wire as the counter electrode. A 0.2 M Na_2SO_4 solution after deoxidation was used as the electrolyte. The concentration of Co-TAPP was determined to 1.0 mM.

Intracellular ROS analysis. 2,7-Dichlorodihydrofluorescein diacetate (DCHF-DA) was cultured with Co-bacteria for sensitive, one-step fluorometric to detect intracellular ROS in live cells within 1-hour incubation. The analysis was performed 24 hours after the illuminated in nitrogen fixation medium. The Co-bacteria in the dark was as the control. The samples were observed *via* a laser scanning confocal microscope (Leica TCS SP8 STED, λ_{ex} = 488/λ_{em} = 530 nm).

Intracellular oxygen analysis. Luminescent oxygen sensor tris(4,7-diphenyl-1,10-phenanthroline)ruthenium(II) dichloride complex is a luminescent hypoxic probe and its fluorescence intensity increases as the

concentration of oxygen decreases. This hypoxic probe was cultured with N_2-fixing bacteria for detecting intracellular oxygen within 1-hour incubation. The samples were observed *via* a laser scanning confocal microscope (Leica TCS SP8 STED, λ_{ex} = 450/λ_{em} = 620 nm). The dead N_2-fixing bacteria was as the control.

Section 3 Supplementary figures

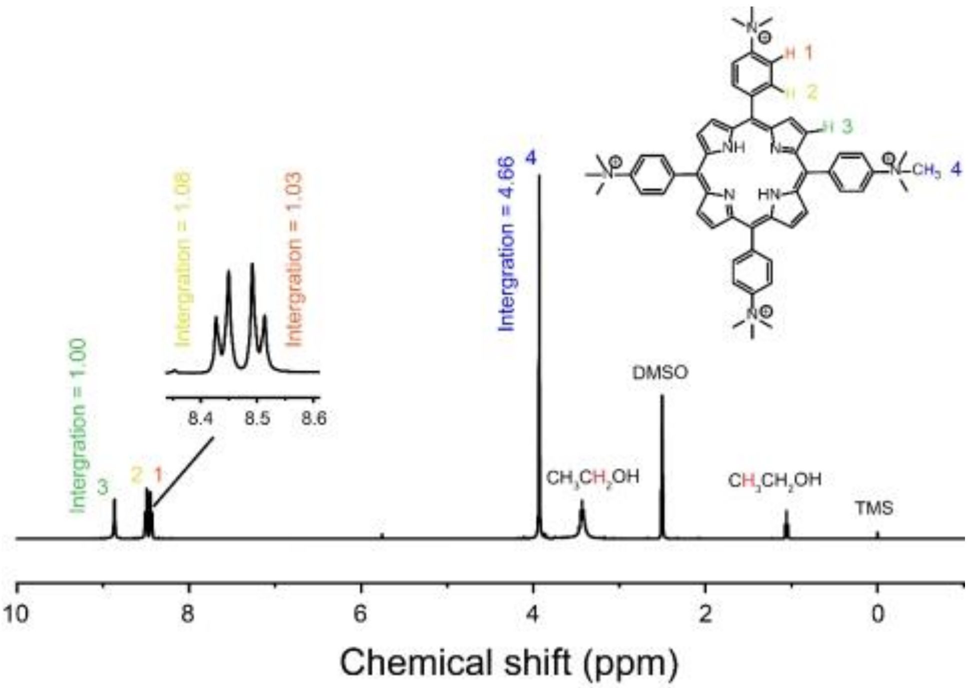

Figure S1 Molecular structure analysis. The ^{1}H NMR spectrum of TTAP and the corresponding molecular structure (solvent, d^6-DMSO).

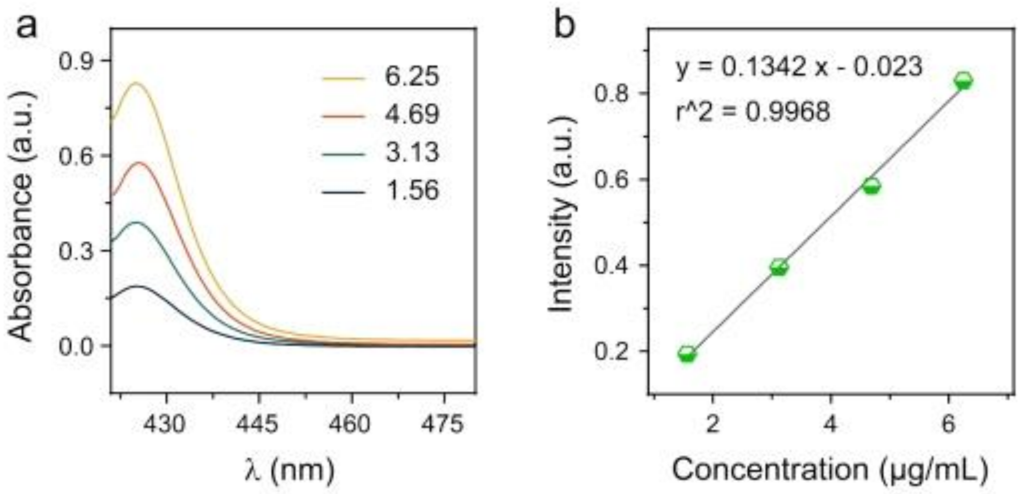

Figure S2 UV-Vis absorbance spectra analysis. (a) UV-Vis absorbance spectra of Co-TTAP in different concentrations. **(b)** The Calibration curve used for estimation of the amount of Co-TTAP.

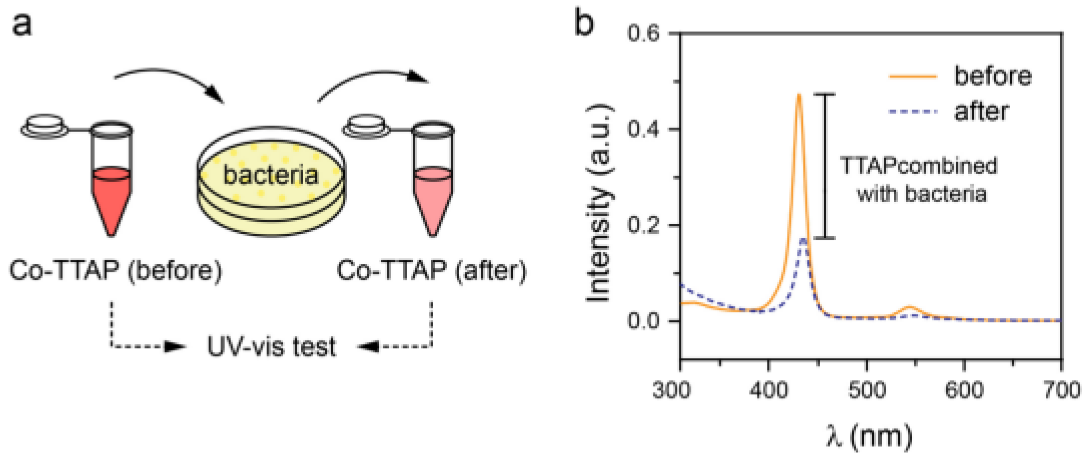

Figure S3 The uptake efficiency analysis of Co-TTAP within N_2-fixing bacteria. **(a)** Schematic diagram of the collection of Co-TTAP solution before and after the combination with N_2-fixing bacteria. **(b)** The UV absorption curves of Co-TTAP before and after the combination with N_2-fixing bacteria.

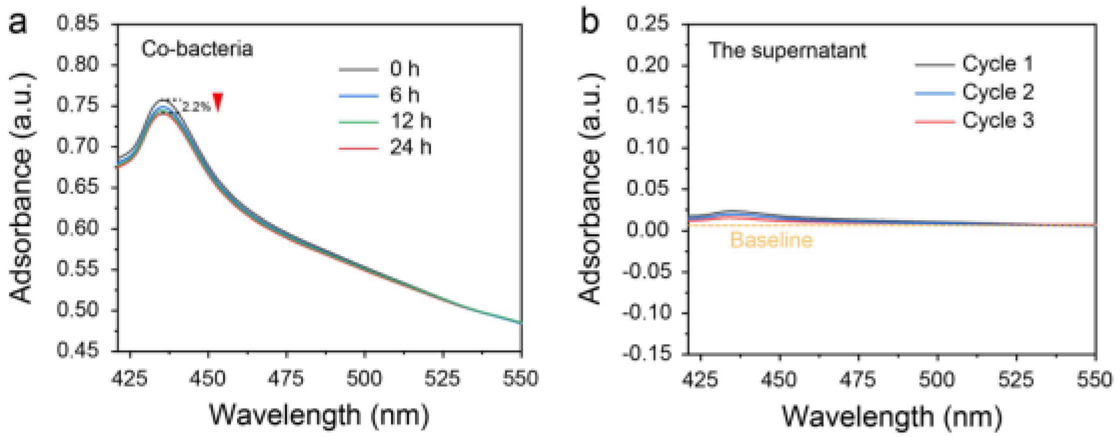

Figure S4 The stability analysis of Co-TTAP within N_2-fixing bacteria. (a) UV-vis absorption spectra of Co-bacteria after soaking different time in PBS. **(b)** UV-vis absorption spectra of the supernatant that was obtained by centrifuging the PBS solution of Co-bacteria. Co-bacteria are soaked in fresh PBS for 6 hours each time.

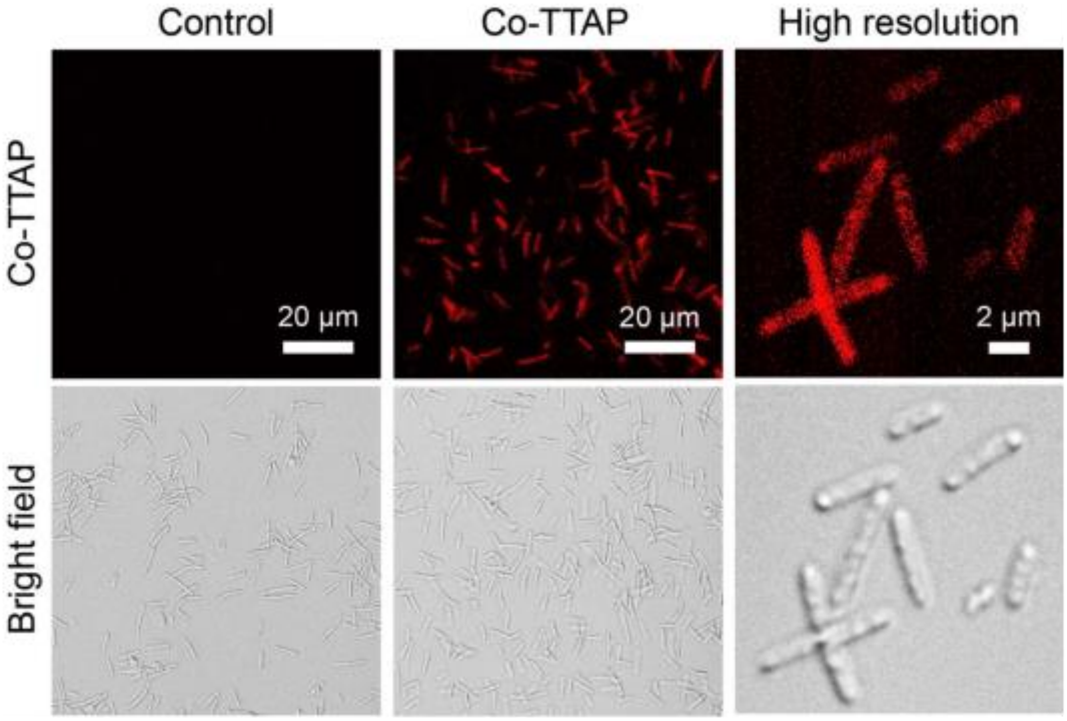

Figure S5 The confocal fluorescence images of bare N_2-fixing bacteria and Co-bacteria (λ_{ex} = 405/λ_{em} = 640 nm). Left, bare N_2-fixing bacteria. Middle, Co-bacteria. Right, high-resolution confocal fluorescence images of Co-bacteria. Top, fluorescence field. Bottom, bright field.

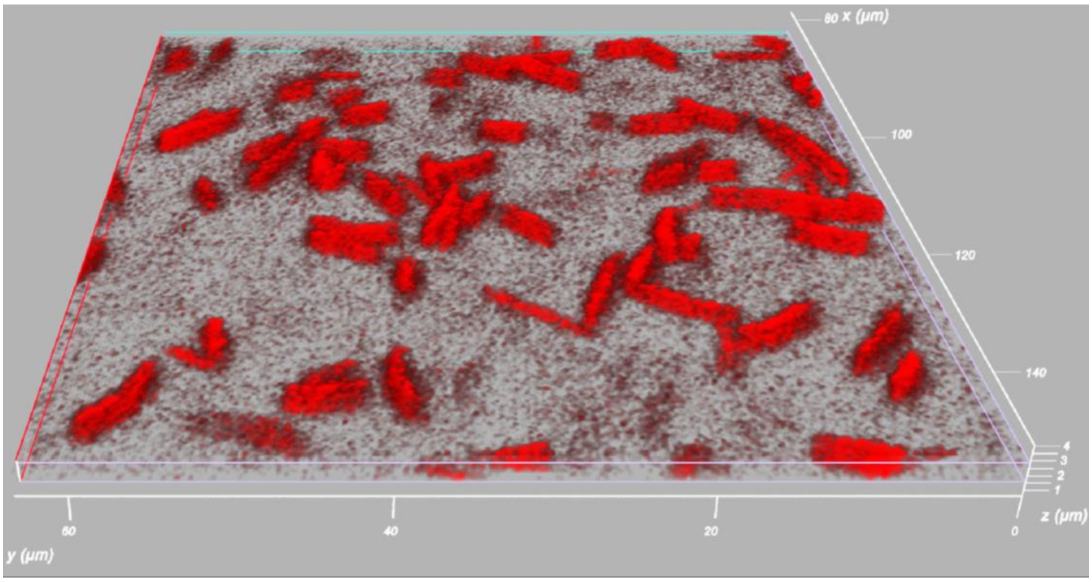

Figure S6 The 3D confocal fluorescence image of Co-bacteria (λ_{ex} = 405/λ_{em} = 640 nm).

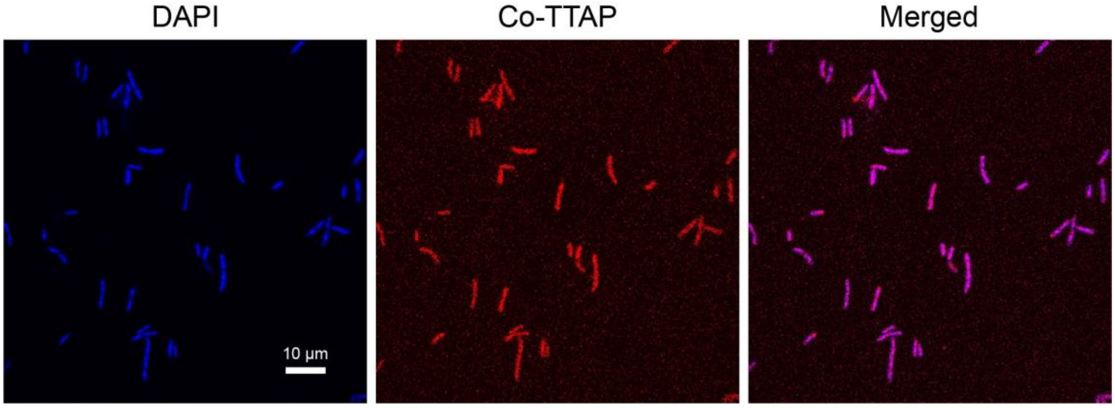

Figure S7 The distribution of Co-TTAP within N_2-fixing bacteria (λ_{ex} = 405/λ_{em} = 640 nm). Confocal fluorescence images showing intracellular DNA and Co-TAPP distribution. The DNA was stained with DAPI (λ_{ex} = 405/λ_{em} = 440 nm).

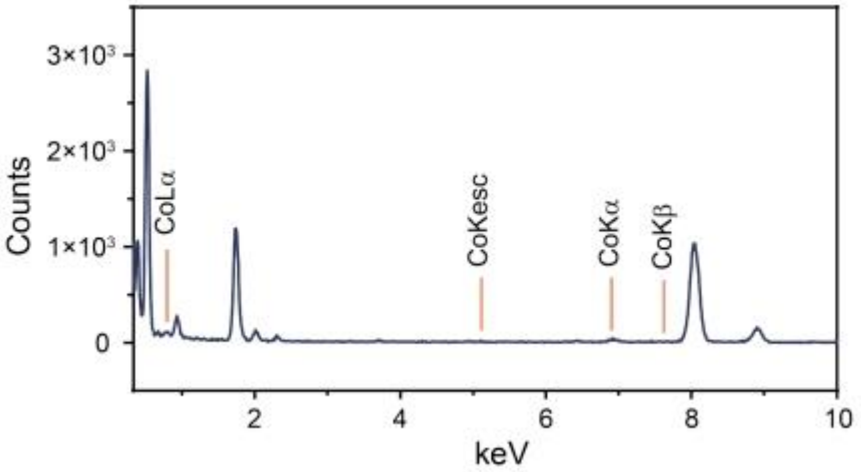

Figure S8 The TEM-EDS analysis of Co-bacteria. TEM-EDS clearly showed the incorporation of Co-TAPP into N_2-fixing bacteria.

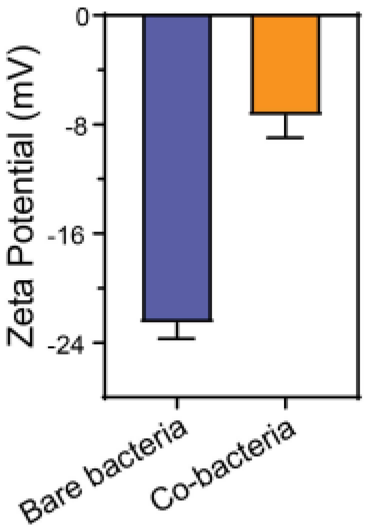

Figure S9 Surface potential analysis. The zeta potential of bare N_2-fixing bacteria and Co-bacteria.

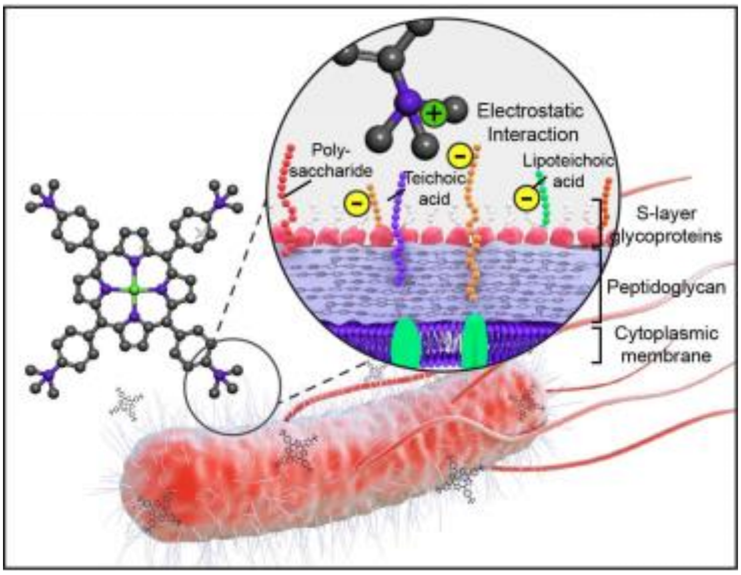

Figure S10 The surface structure interaction analysis. The interactions between the surface structures of N_2-fixing bacteria and Co-TAPP.

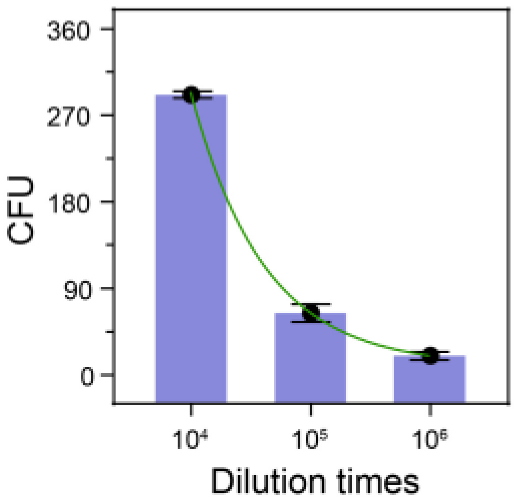

Figure S11 Colony forming unit (CFU) assay of Co-bacteria. The CFU of Co-bacteria (OD600 = 0.3) after diluted by different multiples.

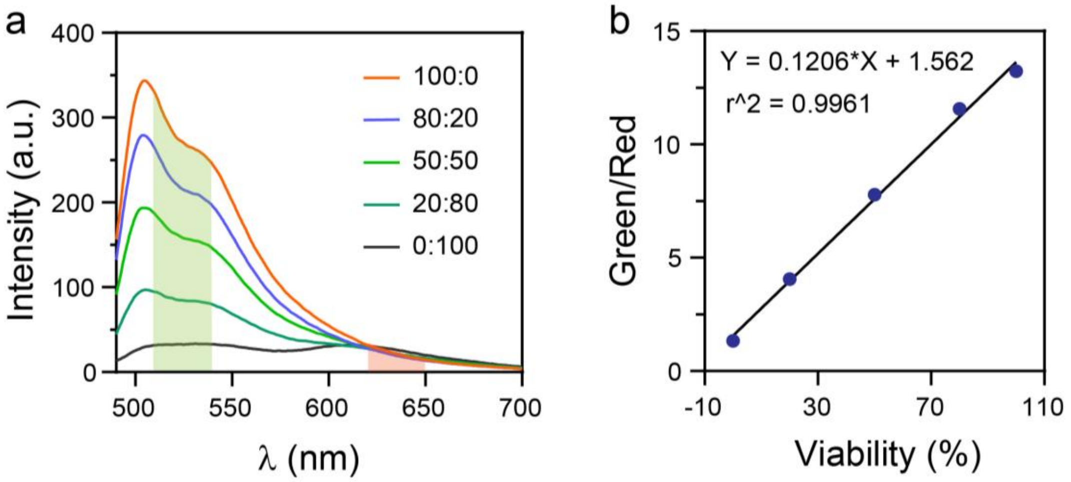

Figure S12 (a) Fluorescence spectra of suspensions of various proportions of live and isopropyl alcohol-killed *Paenibacillus azotofixans* were obtained. (b) Integrated intensities of the green (510-540 nm) and red (620-650 nm) emission were acquired, and the green/red fluorescence ratios (Ratio G/R) were calculated for each proportion of live/dead *Paenibacillus azotofixans*. The line is a least-squares fit of the relationship between % live bacteria (x) and Ratio G/R (y). The live/dead assay to support the viability test was performed by using the Mycolight™ Bacterial Viability Assay Kit that can provide two-color fluorescence assay of bacterial viability in bacterial cell. The kit utilizes the mixture of our green fluorescent nucleic acid stain MycoLight™ Green and the red-fluorescent nucleic acid stain propidium iodide. When used alone, the MycoLight™ Green stain generally labels all bacteria (live and dead) in a population. In contrast, propidium iodide penetrates only bacteria with damaged membranes, causing a reduction in the MycoLight™ Green stain fluorescence when both dyes are present. The stained bacterial cells were analyzed by the fluorescent microplate reader.

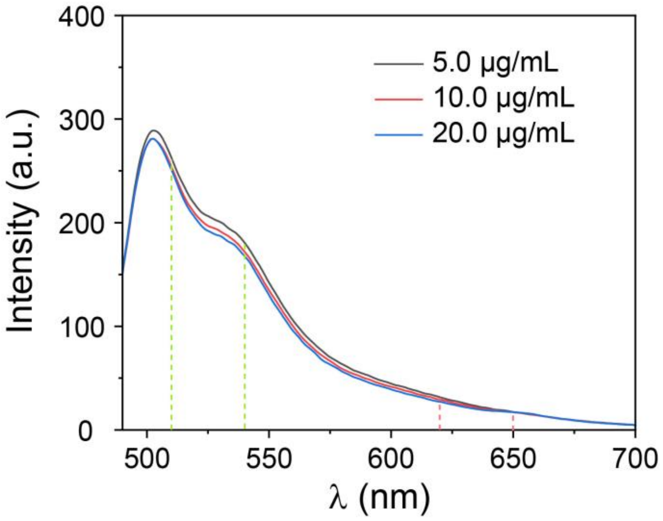

Figure S13 Fluorescence spectra of suspensions of Co-bacteria. The N_2-fixing bacteria were co-cultured with the different concentrations of Co-TAPP (including 5, 10 and 20 μg/mL).

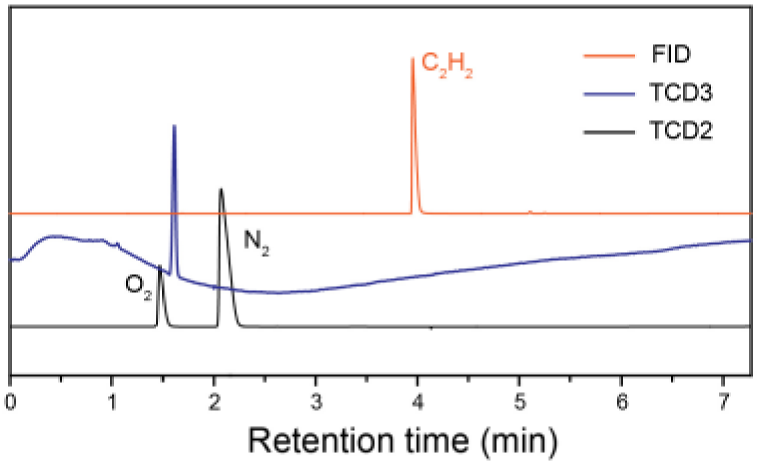

Figure S14 Co-TAPP was used for the acetylene reduction assay. The gas chromatography analysis of the reaction product of Co-TAPP *via* the acetylene reduction assay.

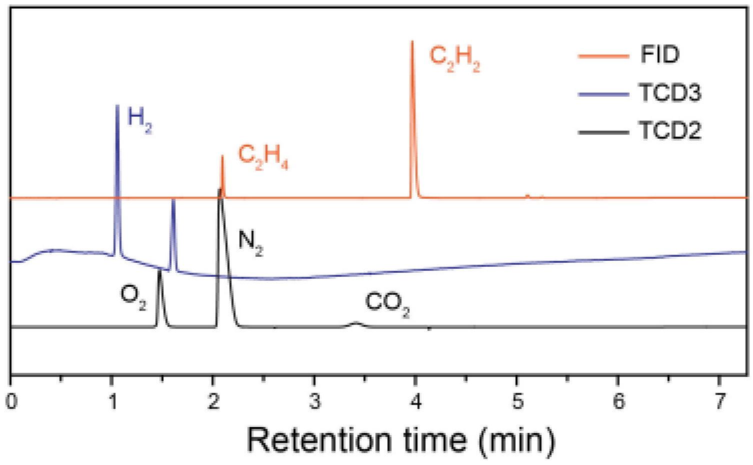

Figure S15 Bare N_2-fixing bacteria were used for the acetylene reduction assay. The gas chromatography analysis of the reaction product of N_2-fixing bacteria *via* the acetylene reduction assay.

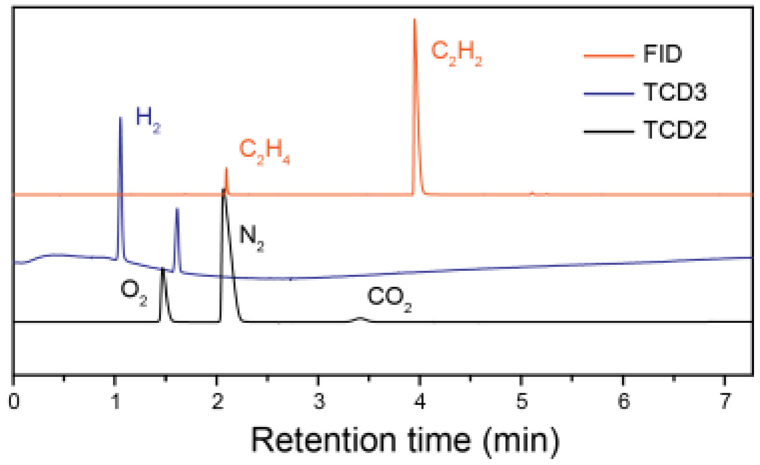

Figure S16 Co-bacteria were used for the acetylene reduction assay. The gas chromatography analysis of the reaction product of Co-bacteria *via* the acetylene reduction assay.

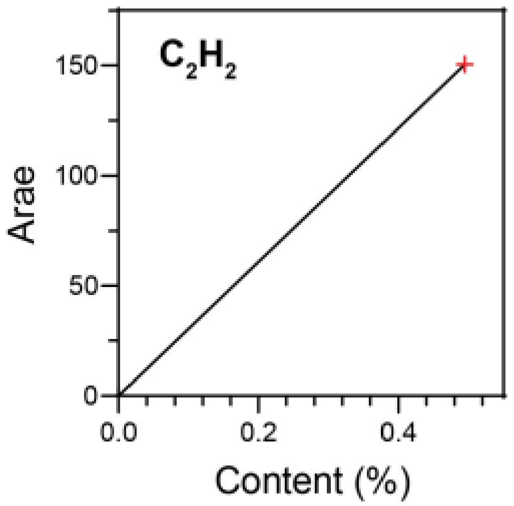

Figure S17 Gas chromatography analysis. The standard calibration curve of acetylene (C_2H_2).

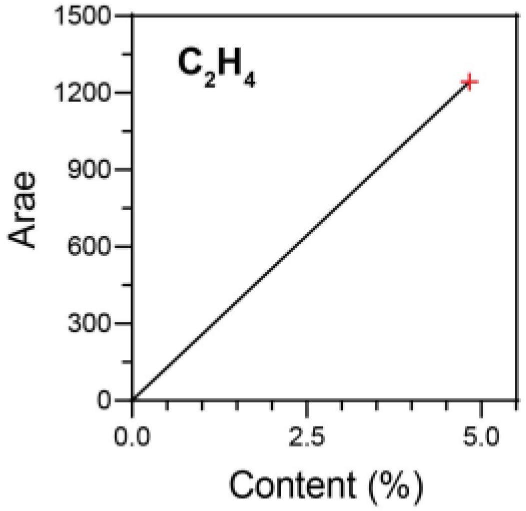

Figure S18 Gas chromatography analysis. The standard calibration curve of ethylene (C_2H_4).

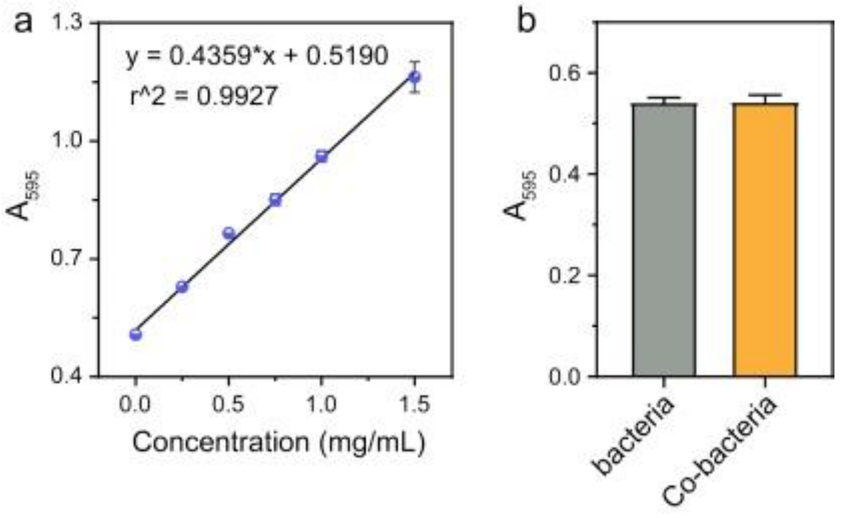

Figure S19 Total protein content analysis of N_2-fixing bacteria and Co-bacteria for evaluating quantitative nitrogenase activity. (a) Protein standard curve determined by Bradford protein assay kit. **(b)** In the Bradford assay, the absorbance of bacterial protein at 595 nm. According to the Bradford method, the protein content of the control bacteria and the bacteria after photocatalysis collected are 0.1019 and 0.1045 mg, respectively.

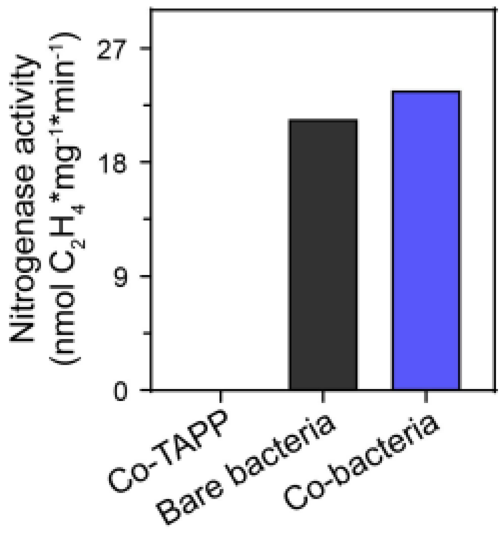

Figure S20 Nitrogenase activity analysis. The nitrogenase activity of Co-TAPP, N_2-fixing bacteria and Co-bacteria.

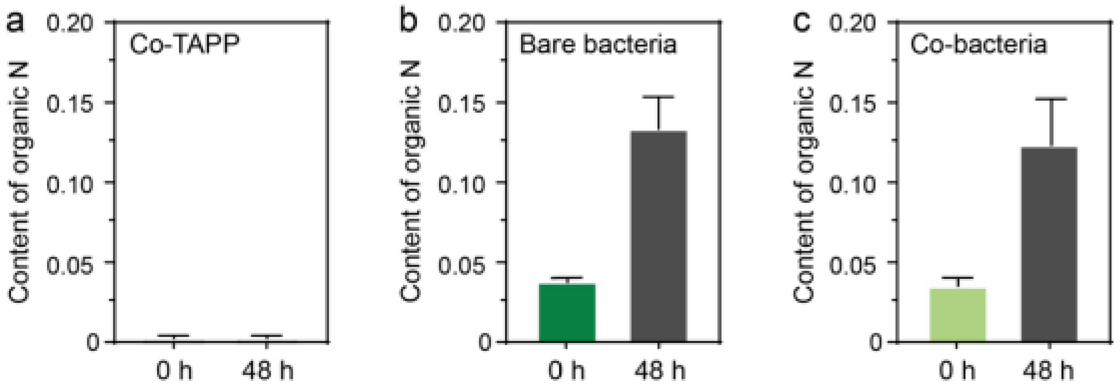

Figure S21 Total organic nitrogen content analysis. **(a)** Organic nitrogen content of Co-TAPP. **(b)** Organic nitrogen content of bare N_2-fixing bacteria. **(c)** Organic nitrogen content of Co-bacteria.

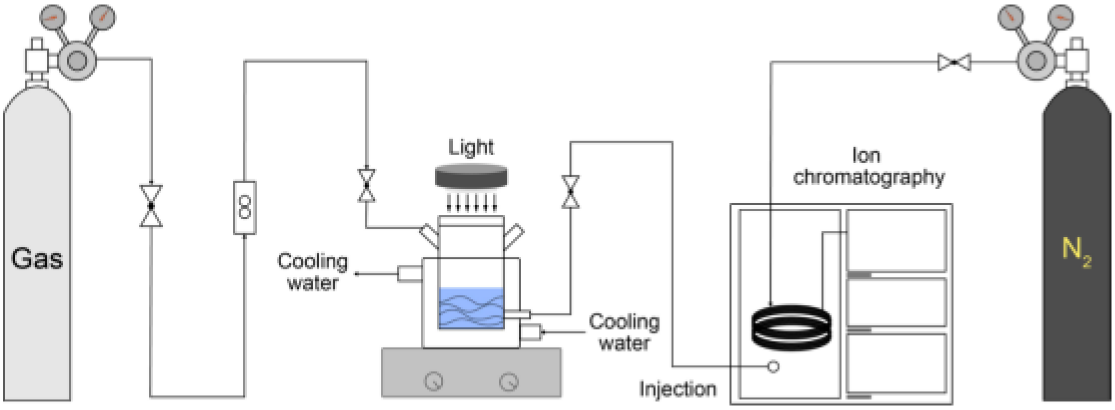

Figure S22 Photocatalytic device. Schematic diagram of photocatalytic CO_2 reduction system.

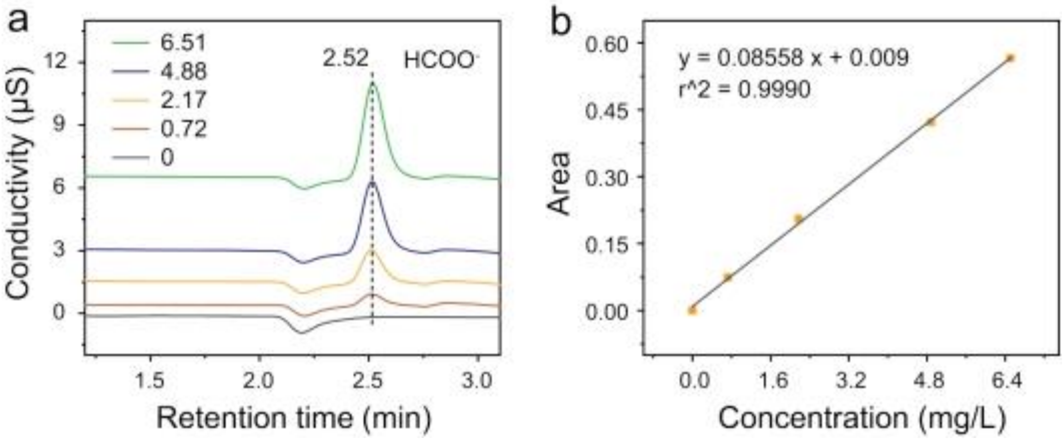

Figure S23 Quantitative analysis of $HCOO^-$ by ion chromatography. **(a)** Ion chromatograms of $HCOO^-$ with different concentrations. **(b)** The standard curve of $HCOO^-$ showed that the integral area of the ion chromatogram has a linear relationship with the corresponding concentration.

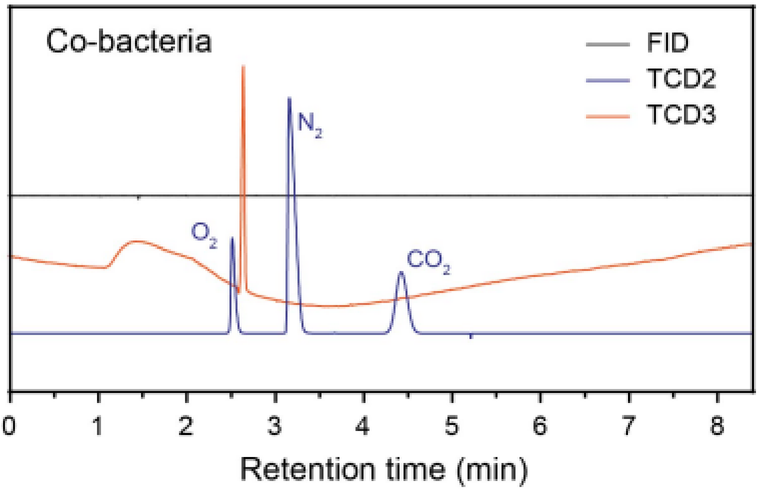

Figure S24 Co-bacteria were used for photocatalytic CO_2 reduction tests. The gas chromatography analysis showed the composition of the gas atmosphere in Co-bacteria photocatalytic system.

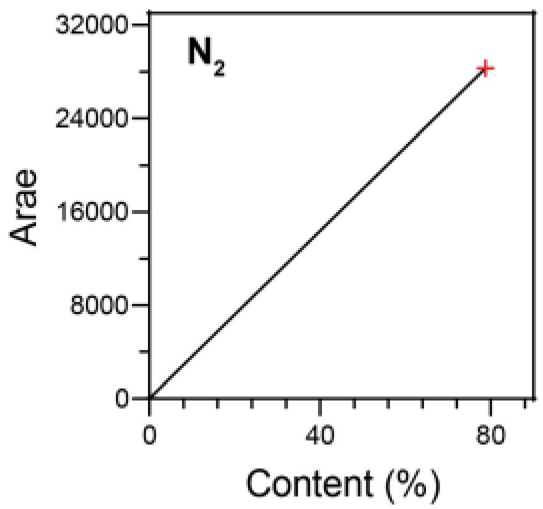

Figure S25 Gas chromatography analysis. The standard calibration curve of nitrogen (N_2).

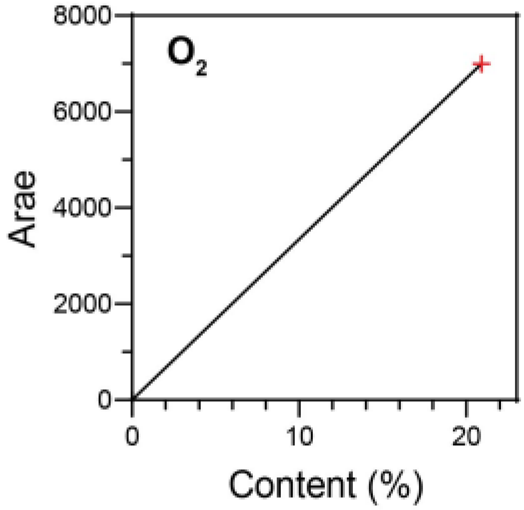

Figure S26 Gas chromatography analysis. The standard calibration curve of oxygen (O_2).

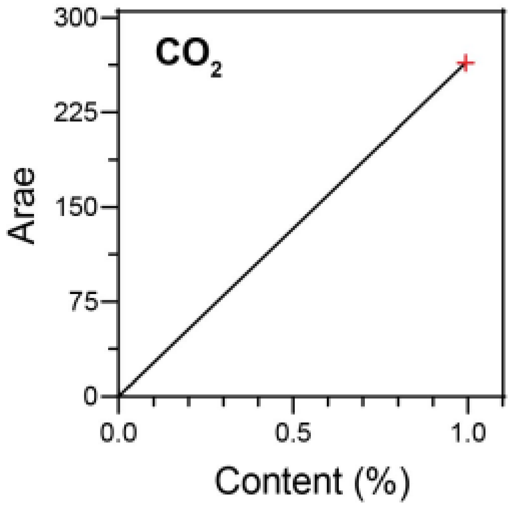

Figure S27 Gas chromatography analysis. The standard calibration curve of carbon dioxide (CO_2).

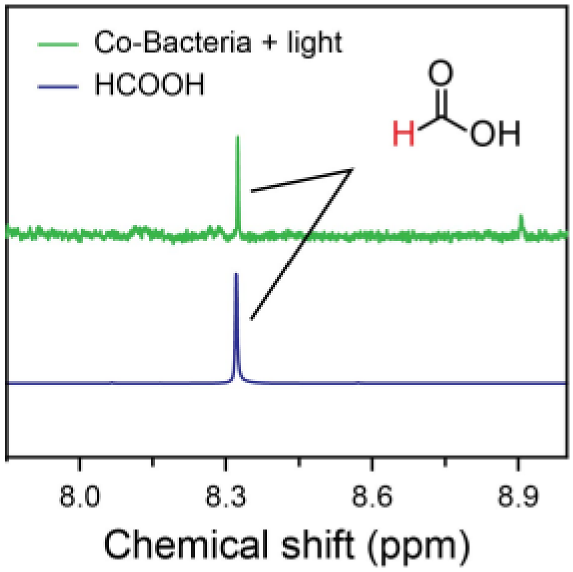

Figure S28 ^{1}H NMR spectrum of liquid product from CO_2 photoreduction by Co-bacteria for 24 hours under light irradiation (solvent, heavy water). Green, liquid product. Blue, standard sample. The sample was collected by distillation and condensation.

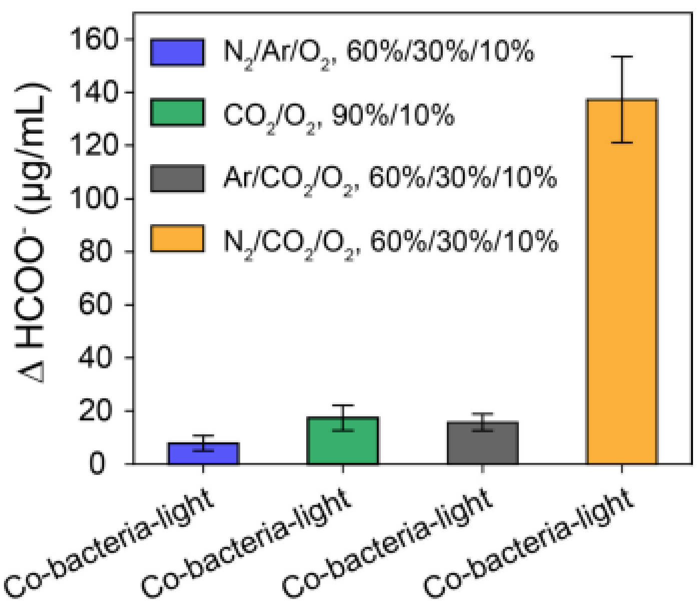

Figure S29 The photocatalytic tests of Co-bacteria were performed under different mixed gas atmospheres.

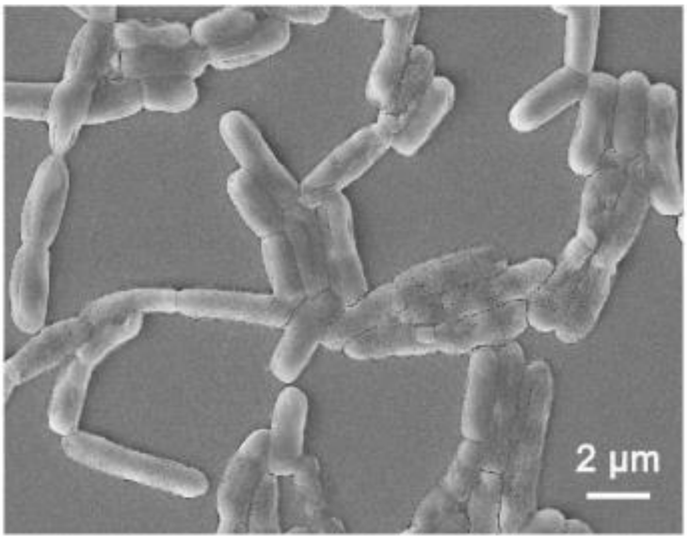

Figure S30 After the photocatalytic reaction, SEM image showed the intact morphology of Co-bacteria.

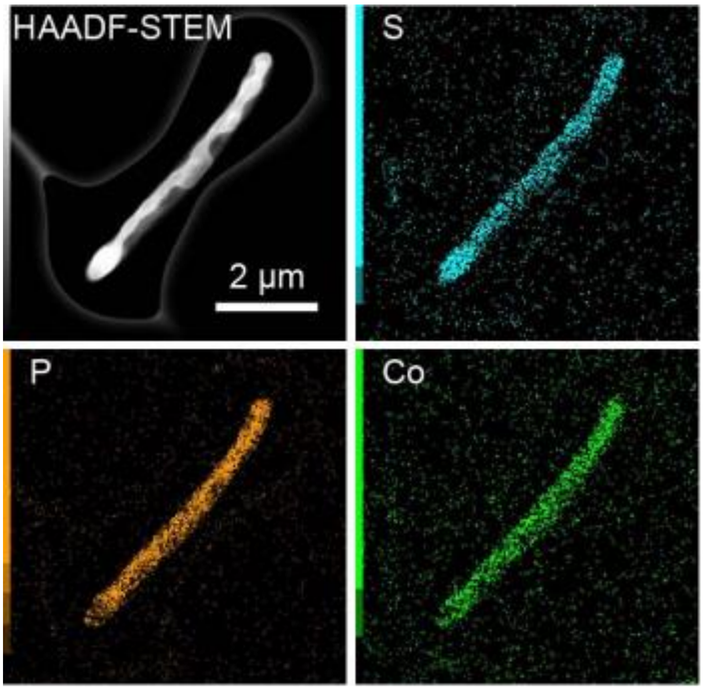

Figure S31 After the photocatalytic reaction, HAADF-STEM image and EDS mapping of Co-bacteria.

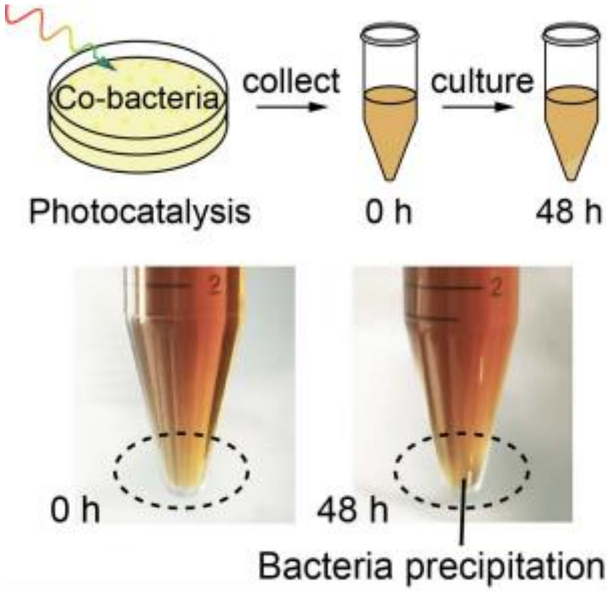

Figure S32 After the photocatalytic reaction, the photographs of the collected Co-bacteria were cultured in a heterotrophic medium for 0 h and 48 h.

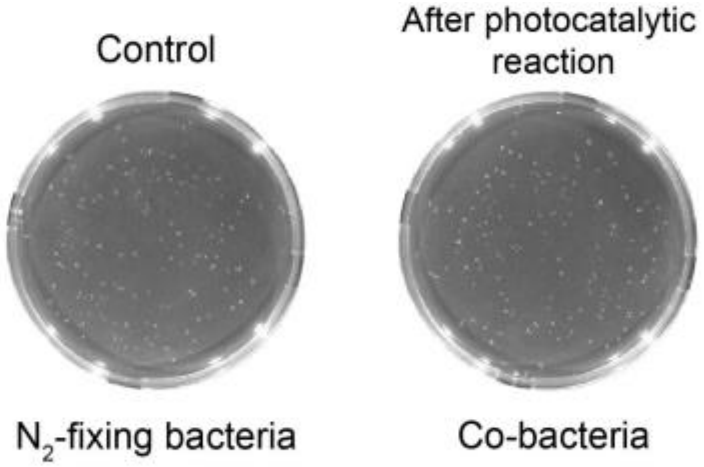

Figure S33 The photographs of colonies grown from the re-cultured bare N_2-fixing bacteria (left) and Co-bacteria (right) after photocatalytic reactions.

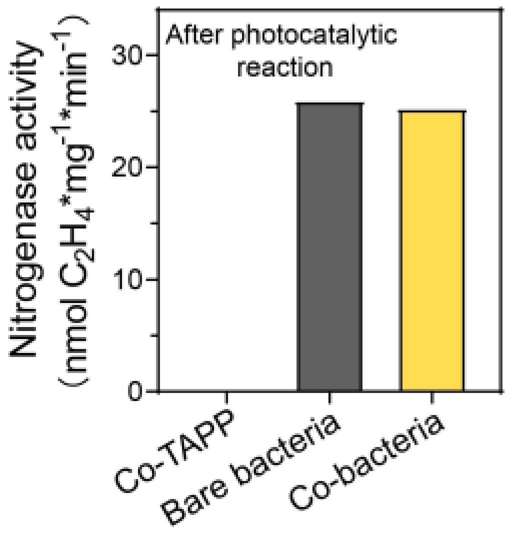

Figure S34 Nitrogenase activity analysis after photocatalytic reaction. The nitrogenase activity of Co-TAPP, N_2-fixing bacteria and Co-bacteria after photocatalytic reaction.

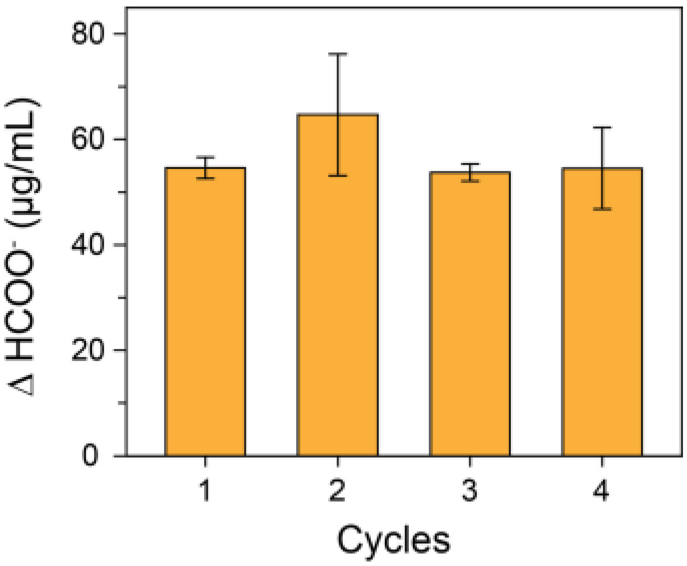

Figure S35 $HCOO^-$ generation through a 4-day test using Co-bacteria under visible light irradiation.

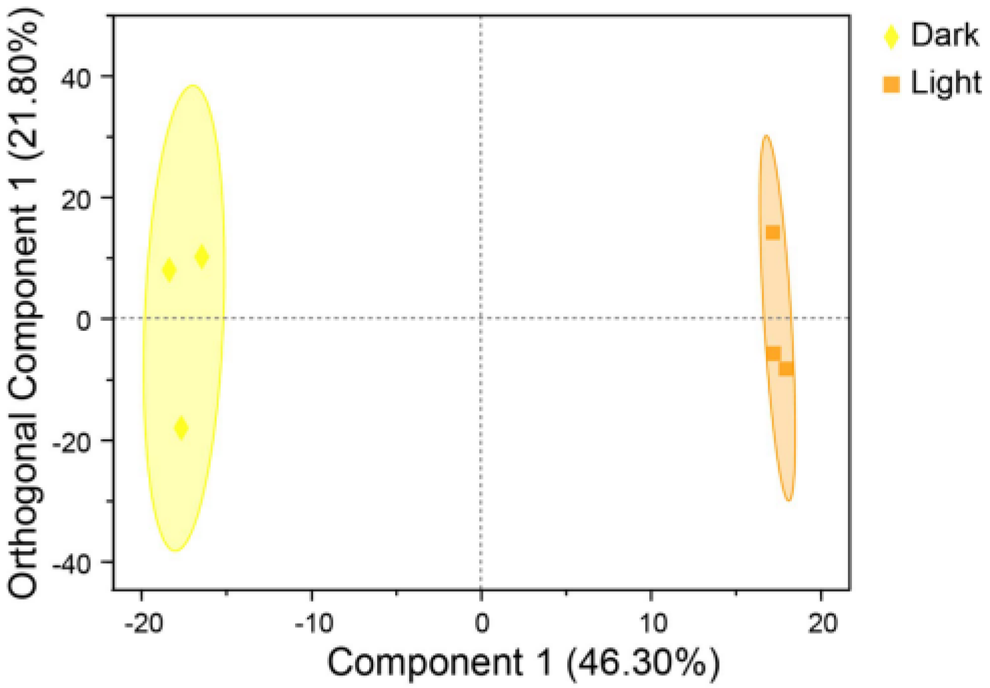

Figure S36 Orthogonal partial least squares discriminant analysis (OPLS-DA) of differential metabolites are used to assess the differences between dark groups and light groups.

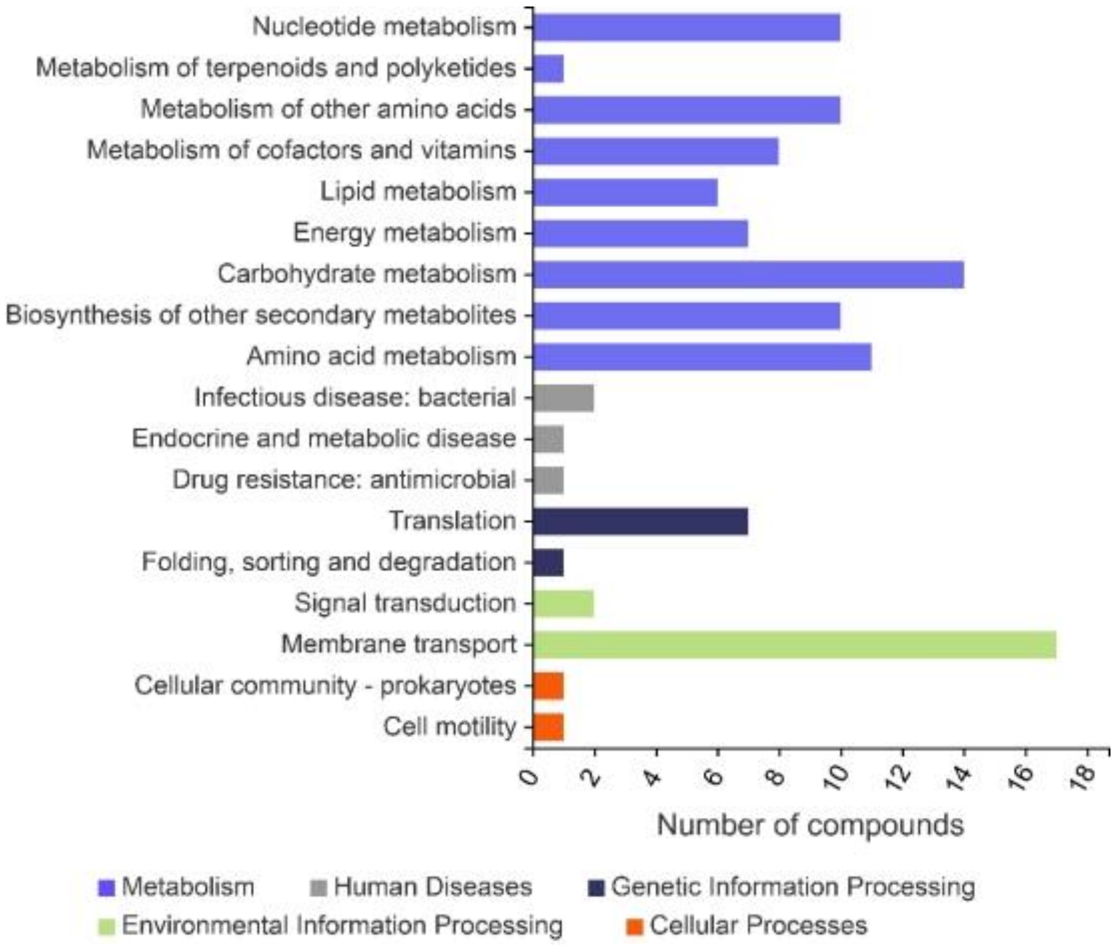

Figure S37 Histogram of functional pathway analysis for the differential metabolites between dark groups and light groups.

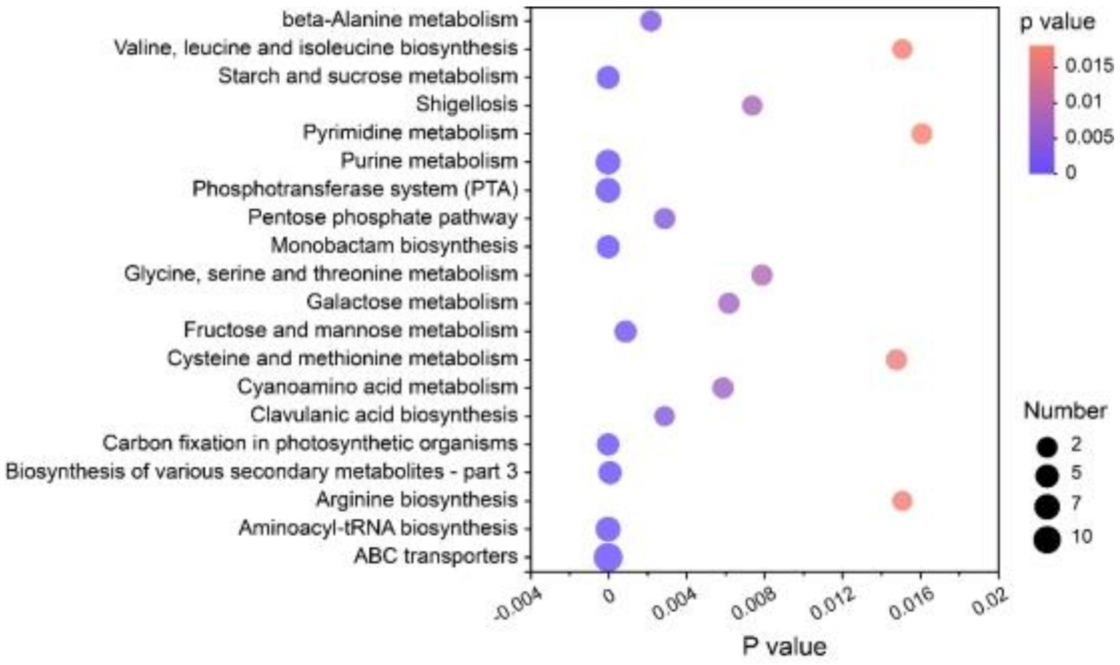

Figure S38 Bubble chart of the KEGG pathway enrichment analysis for the differential metabolites between dark groups and light groups.

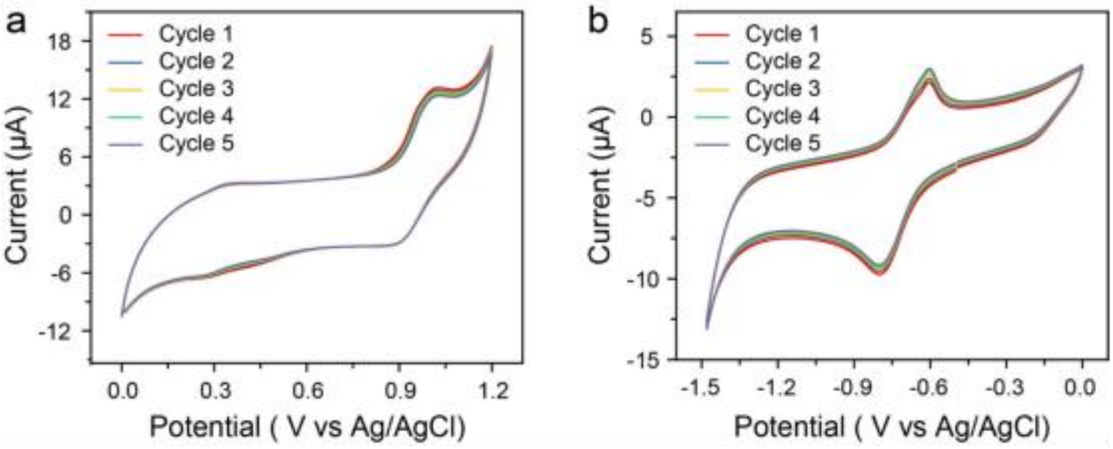

Figure S39 Cyclic Voltammetry tests for Co-TAPP. (a) Oxidation potential scanning (Co-TAPP, 1.0 mM). **(b)** Reduction potential scanning (Co-TAPP, 1.0 mM).

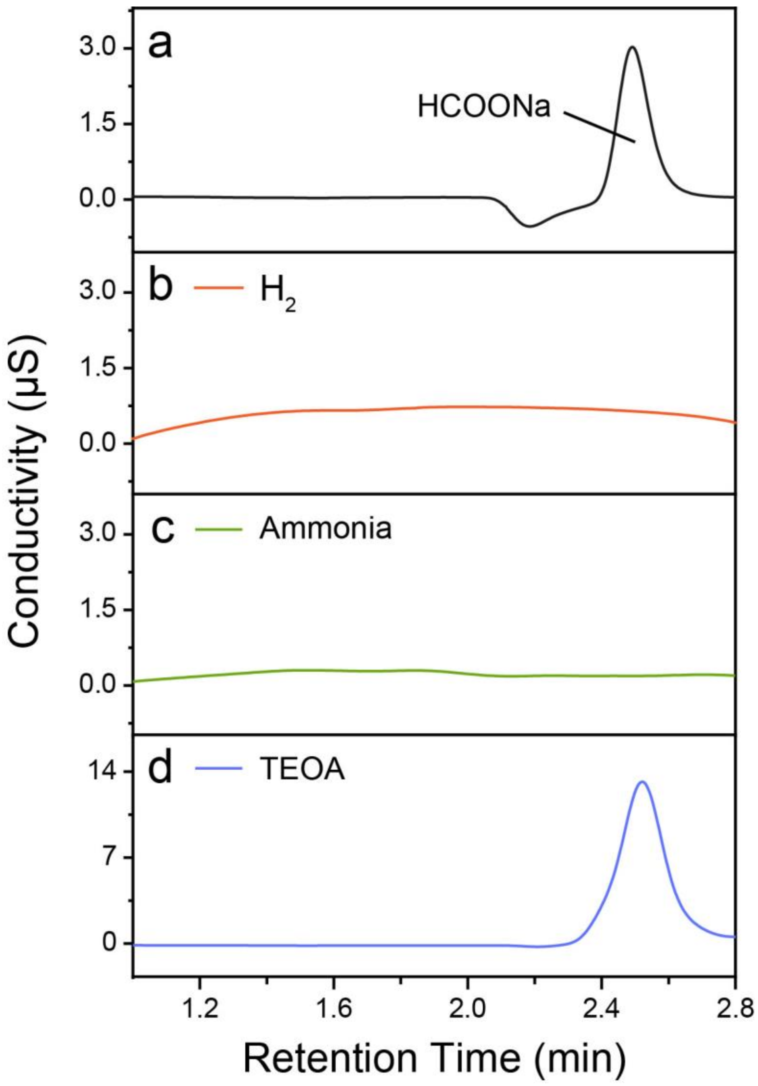

Figure S40 The ion chromatogram for photocatalytic CO_2 reduction tests of Co-TAPP by using different electron donors. **(a)** The standard sample of $HCOO^-$. **(b)** TAPP H_2 as sacrificial electron donors (using the mixed gas that consists of 90% CO_2 and 10% H_2). **(c)** Ammonia as sacrificial electron donors (ammonia, 50 mM). **(d)** Triethanolamine (TEOA) as sacrificial electron donors (TEOA, 50 mM).

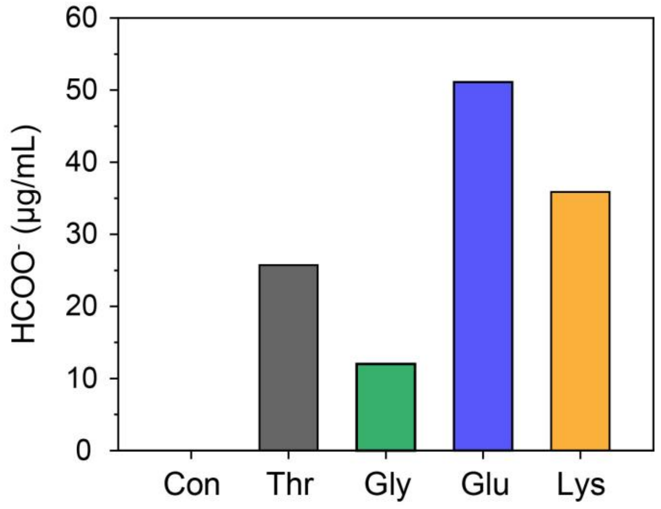

Figure S41 Photocatalytic CO_2 reduction tests of Co-TAPP were performed by using different amino acids as electron donors. No addition of amino acids was used as a control group (Con).

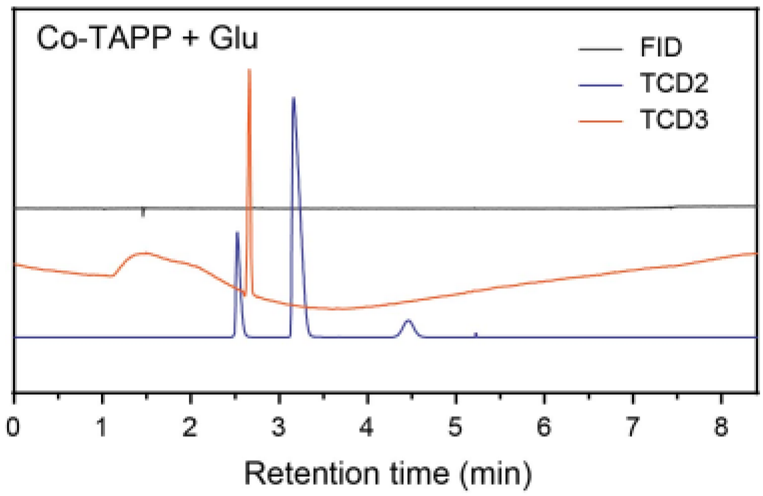

Figure S42 Co-TAPP were used for photocatalytic CO_2 reduction tests. The gas chromatography analysis showed the composition of the gas atmosphere in Co-TTAP photocatalytic system.

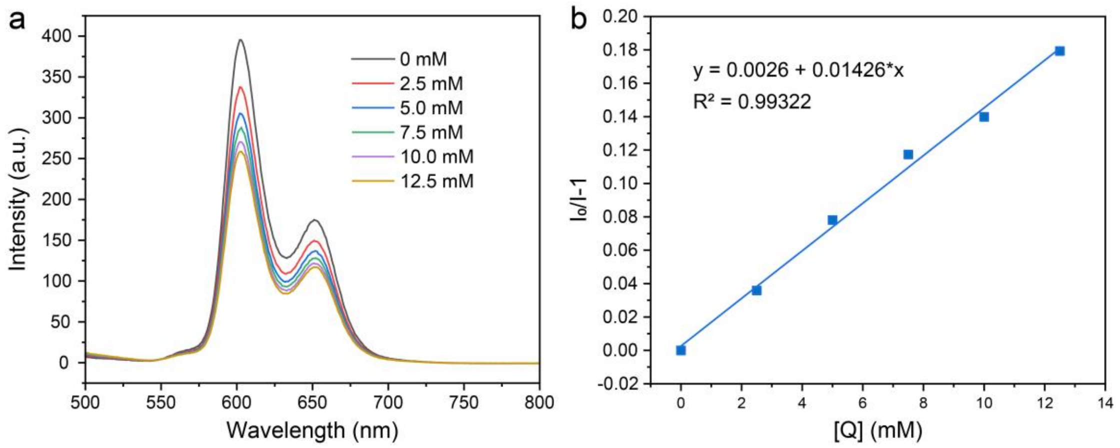

Figure S43 (a) Fluorescence reductively quenched spectrogram of molecular photocatalyst Co-TAPP by using glutamic acid as quencher at 288 K (Excitation wavelength, 415 nm). (b) Linear fitting of the steady-state fluorescence quenching spectrogram at 288 K. The quenching rate constant is calculated according to the Stern-Volmer equation: $I_0/I - 1 = K_q*\tau_0*[Q]$. Here, I_0 is fluorescence intensity without quencher and I is fluorescence intensity with quencher. K_q, τ_0 and [Q] refer to the quenching rate constant, fluorescence lifetime constant and quencher concentration, respectively. Plotting $[I_0/I - 1]$ versus quencher concentration yields a linear relationship, then the slope of the line is $K_q*\tau_0$ value.

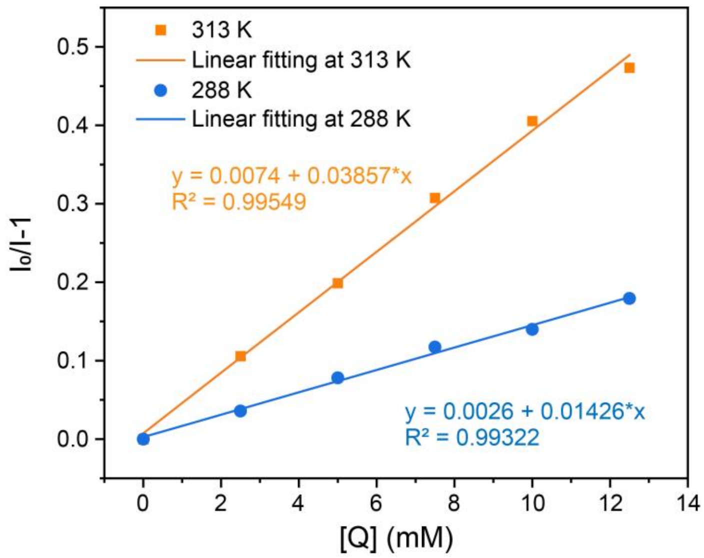

Figure S44 Stern-Volmer curves of molecular photocatalyst Co-TAPP by using glutamic acid as quencher at different temperature. At 288 K, the linear fit equation of steady-state fluorescence quenching was determined to be y = 0.01426x + 0.0026, and then the K_q value is calculated as 8.80 $\times$ 10^9 M^{-1} s^{-1}. At 313 K, the linear fit equation was determined to be y = 0.03857x + 0.0074, and the K_q value is calculated as 2.50 $\times$ 10^{10} M^{-1} s^{-1}. It was found that the quenching rate constant increases gradually with the increase of temperature, indicating that the process is dynamic.

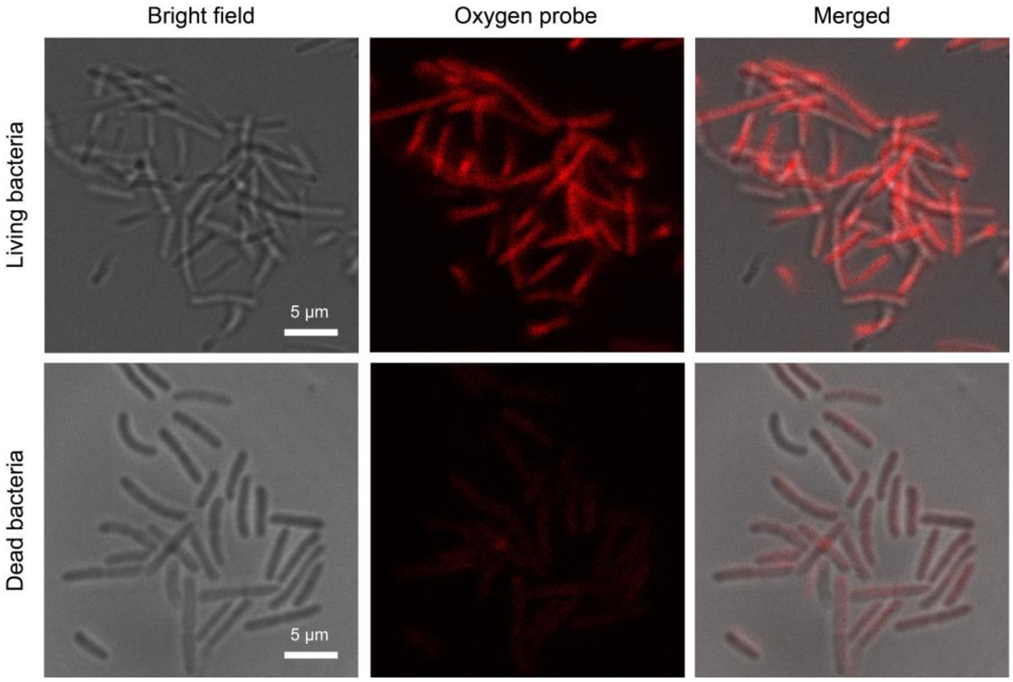

Figure S45 Intracellular oxygen analysis of N_2-fixing bacteria. (λ_{ex} = 450/λ_{em} = 620 nm). Top, living N_2-fixing bacteria; Bottom, dead N_2-fixing bacteria.

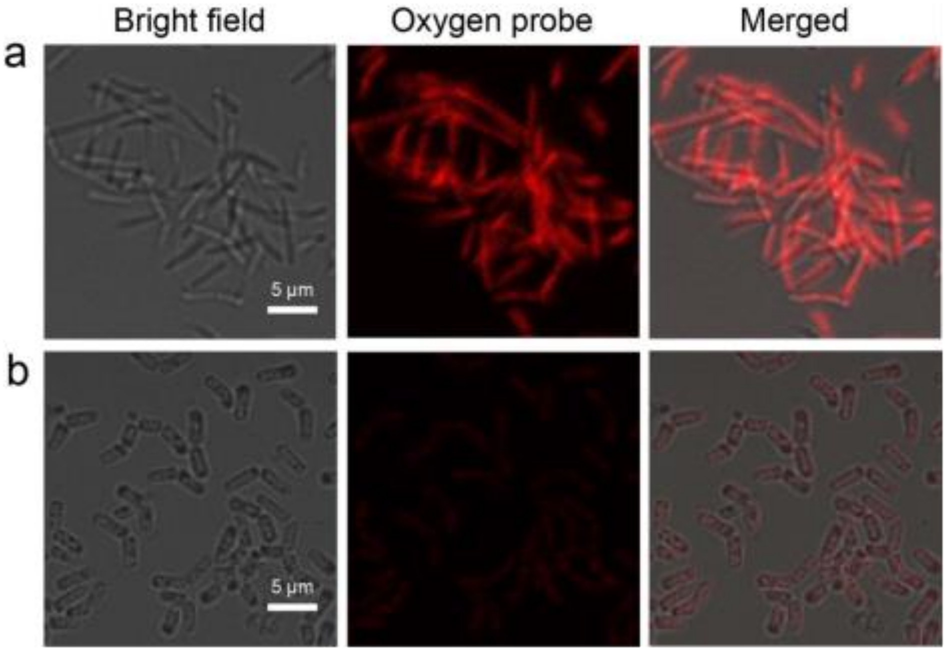

Figure S46 Intracellular oxygen analysis of different bacteria (λ_{ex} = 450/λ_{em} = 620 nm). (a) Live N_2-fixing bacteria; **(b)** Aerobic bacteria without nitrogenase.

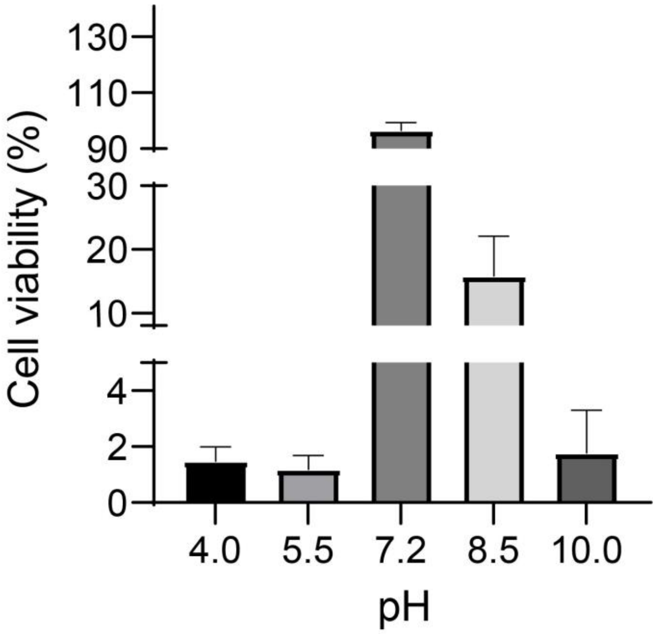

Figure S47 The effects of different pH on the cell viability of biohybid system. The cell viability of N_2-fixing bacteria incorporating Co-TTAP was determined by counting colony-formed units (CFU).

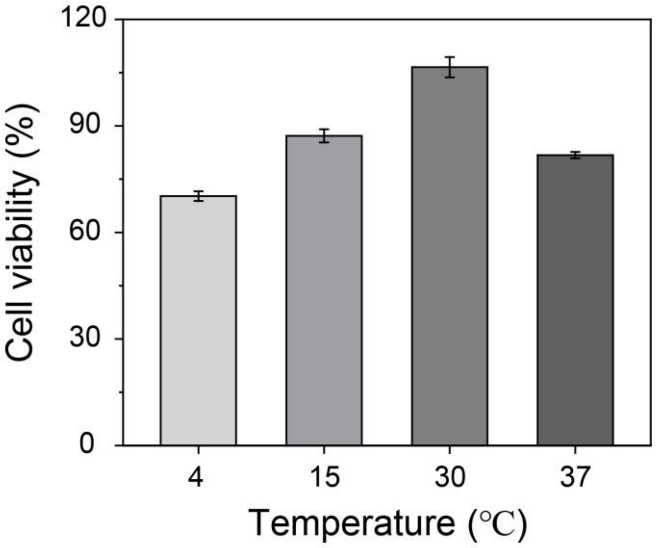

Figure S48 The effects of different temperatures on the cell viability of biohybid system.

Section 4 Supplementary Tables

Table S1. Co-TAPP was used for the acetylene reduction assay. The gas chromatography analysis of the reaction product of Co-TAPP *via* the acetylene reduction assay.

Retention time (min)	Detector	Peak area	Normalized content (%)	Gas name
0.844	FID	-	0	C6+
1.079	TCD3	-	0	Hydrogen
1.498	TCD2	6668.5625	20.2004	Oxygen
1.719	FID	8.0528e-1	0.0063	Methane
1.898	FID	-	0	Ethane
2.104	TCD2	2.7008e4	77.0979	Nitrogen
2.125	FID	-	0	Ethylene
2.521	FID	-	0	Propane
3.303	FID	-	0	Propylene
3.475	TCD2	37.7444	0.1342	Carbon dioxide
3.679	FID	-	0	Isobutane
3.836	FID	-	0	N-butane
3.947	FID	-	0	Allene
4.021	FID	789.8530	2.5490	Acetylene
4.631	FID	-	0	Trans-butene
4.714	FID	-	0	N-butene
4.850	FID	-	0	Isobutylene
4.864	FID	-	0	Neopentane
4.967	FID	-	0	Cis-butene

4.986	TCD2	-	0	Carbon monoxide
5.196	FID	5.5341	0.0088	Isopentane
5.341	FID	1.9766	0.0034	N-pentane
5.598	FID	-	0	1,3-butadiene
5.681	FID	-	0	3-methyl-1-butene
5.826	FID	-	0	Propyne
5.895	FID	-	0	Trans-2-pentene
6.015	FID	-	0	2-methyl-2-butene
6.074	FID	-	0	Pentene
6.115	FID	-	0	2-methyl-1-butene
6.193	FID	-	0	Cis-2-pentene
6.428	FID	-	0	Butyne
6.660	FID	-	0	Isoprene
6.703	FID	-	0	Vinyl acetylene

-, not detected.

Table S2. Bare N_2-fixing bacteria were used for the acetylene reduction assay. The gas chromatography analysis of the reaction product of N_2-fixing bacteria *via* the acetylene reduction assay.

Retention time (min)	Detector	Peak area	Normalized content (%)	Gas name
0.844	FID	-	0	C6+
1.079	TCD3	61.4091	0.3269	Hydrogen
1.498	TCD2	6048.5020	18.3264	Oxygen
1.719	FID	5.3345e-1	0.0042	Methane
1.898	FID	-	0	Ethane
2.104	TCD2	2.6550e4	75.8059	Nitrogen
2.125	FID	54.0809	0.2136	Ethylene
2.521	FID	-	0	Propane
3.303	FID	-	0	Propylene
3.475	TCD2	1087.275	3.8671	Carbon dioxide
3.679	FID	-	0	Isobutane
3.836	FID	-	0	N-butane
3.947	FID	-	0	Allene
4.021	FID	447.5614	1.4447	Acetylene
4.631	FID	-	0	Trans-butene
4.714	FID	-	0	N-butene
4.850	FID	-	0	Isobutylene
4.864	FID	-	0	Neopentane
4.967	FID	-	0	Cis-butene
4.986	TCD2	-	0	Carbon monoxide

5.196	FID	5.1823	0.0081	Isopentane
5.341	FID	1.7820	0.0030	N-pentane
5.598	FID	-	0	1,3-butadiene
5.681	FID	-	0	3-methyl-1-butene
5.826	FID	-	0	Propyne
5.895	FID	-	0	Trans-2-pentene
6.015	FID	-	0	2-methyl-2-butene
6.074	FID	-	0	Pentene
6.115	FID	-	0	2-methyl-1-butene
6.193	FID	-	0	Cis-2-pentene
6.428	FID	-	0	Butyne
6.660	FID	-	0	Isoprene
6.703	FID	-	0	Vinyl acetylene

-, not detected.

Table S3. Co-bacteria were used for the acetylene reduction assay. The gas chromatography analysis of the reaction product of Co-bacteria *via* the acetylene reduction assay.

Retention time (min)	Detector	Peak area	Normalized content (%)	Gas name
0.844	FID	-	0	C6+
1.079	TCD3	73.8270	0.3939	Hydrogen
1.498	TCD2	5887.4385	17.8794	Oxygen
1.719	FID	1.0667	0.0084	Methane
1.898	FID	-	0	Ethane
2.104	TCD2	2.6033e4	74.5029	Nitrogen
2.125	FID	56.1092	0.2221	Ethylene
2.521	FID	-	0	Propane
3.303	FID	-	0	Propylene
3.475	TCD2	1038.1743	3.7010	Carbon dioxide
3.679	FID	-	0	Isobutane
3.836	FID	-	0	N-butane
3.947	FID	-	0	Allene
4.021	FID	1013.7735	3.2799	Acetylene
4.631	FID	-	0	Trans-butene
4.714	FID	-	0	N-butene
4.850	FID	-	0	Isobutylene
4.864	FID	-	0	Neopentane
4.967	FID	-	0	Cis-butene
4.986	TCD2	-	0	Carbon monoxide

5.196	FID	5.5692	0.0089	Isopentane
5.341	FID	2.0295	0.0035	N-pentane
5.598	FID	-	0	1,3-butadiene
5.681	FID	-	0	3-methyl-1-butene
5.826	FID	-	0	Propyne
5.895	FID	-	0	Trans-2-pentene
6.015	FID	-	0	2-methyl-2-butene
6.074	FID	-	0	Pentene
6.115	FID	-	0	2-methyl-1-butene
6.193	FID	-	0	Cis-2-pentene
6.428	FID	-	0	Butyne
6.660	FID	-	0	Isoprene
6.703	FID	-	0	Vinyl acetylene

-, not detected.

Table S4. Co-bacteria were used for photocatalytic CO_2 reduction tests. The gas chromatography analysis showed the composition of the gas atmosphere in Co-bacteria photocatalytic system.

Retention time (min)	Detector	Peak area	Normalized content (%)	Gas name
0.844	FID	-	0	C6+
1.079	TCD3	-	0	Hydrogen
1.498	TCD2	4809.8057	15.3122	Oxygen
1.719	FID	-	0	Methane
1.898	FID	-	0	Ethane
2.104	TCD2	2.0045e4	58.7912	Nitrogen
2.125	FID	-	0	Ethylene
2.521	FID	-	0	Propane
3.303	FID	-	0	Propylene
3.475	TCD2	8649.4580	28.7011	Carbon dioxide
3.679	FID	-	0	Isobutane
3.836	FID	-	0	N-butane
3.947	FID	-	0	Allene
4.021	FID	-	0	Acetylene
4.631	FID	-	0	Trans-butene
4.714	FID	-	0	N-butene
4.850	FID	-	0	Isobutylene
4.864	FID	-	0	Neopentane
4.967	FID	-	0	Cis-butene
4.986	TCD2	-	0	Carbon monoxide

5.196	FID	-	0	Isopentane
5.341	FID	-	0	N-pentane
5.598	FID	-	0	1,3-butadiene
5.681	FID	-	0	3-methyl-1-butene
5.826	FID	-	0	Propyne
5.895	FID	-	0	Trans-2-pentene
6.015	FID	-	0	2-methyl-2-butene
6.074	FID	-	0	Pentene
6.115	FID	-	0	2-methyl-1-butene
6.193	FID	-	0	Cis-2-pentene
6.428	FID	-	0	Butyne
6.660	FID	-	0	Isoprene
6.703	FID	-	0	Vinyl acetylene

-, not detected.

Table S5. Co-TAPP were used for photocatalytic CO_2 reduction tests. The gas chromatography analysis showed the composition of the gas atmosphere in Co-TTAP photocatalytic system (glutamic acid was used as sacrificial electron donor).

Retention time (min)	Detector	Peak area	Normalized content (%)	Gas name
0.844	FID	-	0	C6+
1.079	TCD3	-	0	Hydrogen
1.498	TCD2	6291.1138	19.7950	Oxygen
1.719	FID	-	0	Methane
1.898	FID	-	0	Ethane
2.104	TCD2	2.5381e4	74.2162	Nitrogen
2.125	FID	-	0	Ethylene
2.521	FID	-	0	Propane
3.303	FID	-	0	Propylene
3.475	TCD2	2601.2058	8.6497	Carbon dioxide
3.679	FID	-	0	Isobutane
3.836	FID	-	0	N-butane
3.947	FID	-	0	Allene
4.021	FID	-	0	Acetylene
4.631	FID	-	0	Trans-butene
4.714	FID	-	0	N-butene
4.850	FID	-	0	Isobutylene
4.864	FID	-	0	Neopentane
4.967	FID	-	0	Cis-butene
4.986	TCD2	-	0	Carbon monoxide

5.196	FID	-	0	Isopentane
5.341	FID	-	0	N-pentane
5.598	FID	-	0	1,3-butadiene
5.681	FID	-	0	3-methyl-1-butene
5.826	FID	-	0	Propyne
5.895	FID	-	0	Trans-2-pentene
6.015	FID	-	0	2-methyl-2-butene
6.074	FID	-	0	Pentene
6.115	FID	-	0	2-methyl-1-butene
6.193	FID	-	0	Cis-2-pentene
6.428	FID	-	0	Butyne
6.660	FID	-	0	Isoprene
6.703	FID	-	0	Vinyl acetylene

-, not detected.

Table S6. Comparison of the external sacrificial reagent of previously reported biohybrid systems and our biohybrid system in this work.

Life-unit organism	Photocatalyst	Substrate	Products	**External sacrificial reagent**	Light source	AQE* (%)	Ref
M. thermoacetica	CdS	CO_2	Acetate	**Cysteine**	Xe lamp	2.44	1
M. thermoacetica	$Au_{22}(SG)_{18}$	CO_2	Acetate	**Cysteine**	Xe lamp	2.86	2
S. cerevisiae	InP	DHS	Shikimic acid	**Hexose**	Cold-white	1.58	3
M. thermoacetica	CdS /TiO_2-MnPc	CO_2	Acetate	**Cysteine**	Xe lamp	N. A.	4
R. palustris	CdS	CO_2	PHB	**Cysteine**	N. A.	1.67	5
A. vinelandii	CdS@ZnS	CO_2	Formic acid	**L-ascorbic acid**	400 nm LED	N. A.	6
C. necator	CdS@ZnS	CO_2	Ethylene	**L-ascorbic acid**	400 nm LED	0.6	6
M. thermoacetica	PDI/PFP	CO_2	Acetate	**Cysteine**	$\lambda \geq 420$ nm	1.6	7
E. coli	CdS	H_2O	H_2	**L-ascorbic acid**	Xe lamp	N. A.	8
E. coli	I-HTCC	H^+	H_2	**Cysteine**	700 nm	9.11	9
E. coli	TiO_2	H^+	H_2	**L-ascorbic acid**	420 nm	0.1	10

S. ovata	Si nanowire	CO_2	Acetate	**N. A.**	Sunlight	N. A.	11
Paenibacillus azotofixans	Co-TTAP	CO_2	Formic acid	**N_2 fixtion**	$\lambda \geq 420$ nm	2.25	**This work**

*, Calculated by consumed electron number. N. A., not available. DHS, 3-dehydroshikimic acid; PHB, polyhydroxybutyrate; PDI/PFP, Perylene diimide derivative/poly(fluorene-co-phenylene). Quantum yield is represented by the ratio of electrons used to reduce a substrate to a final product and the total input photon flux as per ref.[1]

References

(S1) Sakimoto, K. K., Wong, A. B. & Yang, P. Self-photosensitization of nonphotosynthetic bacteria for solar-to-chemical production. *Science* **351**, 74-77 (2016).

(S2) Zhang, H. et al. Bacteria photosensitized by intracellular gold nanoclusters for solar fuel production. *Nat. Nanotechnol.* **13**, 900-905 (2018).

(S3) Guo, J. et al. Light-driven fine chemical production in yeast biohybrids. *Science* **362**, 813-816 (2018).

(S4) Sakimoto, K. K., Zhang, S. J. & Yang, P. Cysteine-cystine photoregeneration for oxygenic photosynthesis of acetic acid from CO_2 by a tandem inorganic biological hybrid system. *Nano Lett.* **16**, 5883-5887 (2016).

(S5) Wang, B., Jiang, Z., Yu, J. C., Wang, J. & Wong, P. K. Enhanced CO_2 reduction and valuable C_{2+} chemical production by a CdS-photosynthetic hybrid system. *Nanoscale* **11**, 9296-9301 (2019).

(S6) Ding, Y. et al. Nanorg microbial factories: light-driven renewable biochemical synthesis using quantum dot-bacteria nanobiohybrids. *J. Am. Chem. Soc.* **141**, 10272-10282 (2019).

(S7) Gai, P. *et al*. Solar-powered organic semiconductor-bacteria biohybrids for CO_2 reduction into acetic acid. *Angew. Chem. Int. Ed.* **59**, 7224 -7229 (2020).

(S8) Wei, W. et al. A surface-display biohybrid approach to light-driven hydrogen production in air. *Sci. Adv.* **4**, eaap9253 (2018).

(S9) Xiao, K. *et al*. Interfacing iodine-doped hydrothermally carbonized carbon with *Escherichia coli* through an “add-on” mode for enhanced light-driven hydrogen production. *Adv. Energy Mater.* **11**, 2100291 (2021).

(S10) Honda, Y., Watanabe, M., Hagiwara, H., Ida, S. & Ishihara, T. Inorganic/whole-cell biohybrid photocatalyst for highly effificient hydrogen production from water. *Appl. Catal. B* **210**, 400-406 (2017).

(S11) Liu, C. *et al*. Nanowire-bacteria hybrids for unassisted solar carbon dioxide fixation to value-added chemicals. *Nano Lett.* **15**, 3634-3639 (2015).
